# A simple model captures key characteristics of biological non-deterministic genotype-phenotype maps

**DOI:** 10.1371/journal.pcbi.1014272

**Published:** 2026-05-22

**Authors:** Nora S. Martin

**Affiliations:** CRG (Barcelona Collaboratorium for Modelling and Predictive Biology), Barcelona, ‌‌Spain; Rutgers University: Rutgers The State University of New Jersey, UNITED STATES OF AMERICA

## Abstract

By connecting genotypic mutations to the higher-level phenotypes relevant for selection, genotype-phenotype (GP) maps play a key role in evolution. GP maps are typically investigated using computational models of biophysical phenotypes (for example, RNA secondary structures and simplified models of protein tertiary and quaternary structures), but GP map concepts are relevant beyond these specific models. While there has been significant progress in quantifying GP map properties and their evolutionary implications, this is largely limited to the simplest case, where each genotype corresponds to a single, categorical phenotype. Here, I turn to a more realistic, but also more complex, non-deterministic (ND) treatment, meaning that each genotype generates an ensemble of phenotypes. To provide a tool for tackling the additional complexity of ND GP maps, this paper identifies a tuneable synthetic model that produces an ND GP map reproducing central features of biophysical ND GP maps: phenotypic bias, genetic correlations, a tradeoff between genotypic robustness and evolvability and a non-negative trend between phenotypic robustness and evolvability. These features are reproduced for several alternative models combining additive genotype dependencies with non-linearities, suggesting that few ingredients are needed for these shared features to appear. Moreover, the synthetic ND GP map may be useful as a conceptually and computationally simpler model for addressing open questions about ND GP maps: for simulations linking GP map properties to evolutionary implications, for the development of sampling methods for ND GP maps and for extrapolations.

## Author summary

Genotype-phenotype maps connect genotypic mutations to their phenotypic effect, and are thus important for modelling evolution. Many realistic models of such maps are non-deterministic, meaning that each genotype corresponds to an ensemble of phenotypes rather than to a single, categorical phenotype. This non-determinism, combined with the high number of possible genotypes, means that genotype-phenotype maps are huge datasets, and thus complex to build, to analyse and to use in evolutionary models. To provide a tool for tackling this complexity, I show that a simple model produces a non-deterministic genotype-phenotype map that – despite its simplicity - mirrors important shared features of genotype-phenotype maps derived from biophysical models. Thus, this model is highly suitable as a tool for addressing open questions about non-deterministic genotype-phenotype maps. Moreover, I consider modified versions of the simple model, finding that several of them reproduce the shared features of biophysical genotype-phenotype maps. This implies that, rather than being special properties of biophysical models, these shared features easily emerge from few ingredients.

## 1. Introduction

Variation through random mutations is a central component of models of evolutionary processes [3]. Since variation at the phenotypic level is produced by mutations on the genotypic level, a *genotype-phenotype (GP) map* is needed to model variation quantitatively. GP maps can be characterised by a set of quantitative features [4] such as phenotypic frequencies and evolvabilities. These features can be computed for any given GP map and thus facilitate a comparison of GP maps describing different phenotypes on the molecular scale and beyond and thus highlight shared GP map properties whose evolutionary implications are relevant beyond ‌‌a single GP map [3,4].

**Fig 1:**
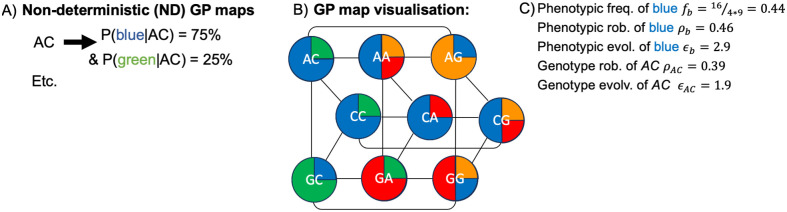
Non-deterministic (ND) GP maps and their characterisation (see [1] for a similar schematic): **A)** An ND GP map is a dataset, where each possible genotype (here sequences of length *L* = 2 and an alphabet size *K* = 3 for simplicity) maps to a probability distribution over phenotypes, here represented by four colours. **B)** A convenient representation of a GP map is as a network: each genotype is a node, each node has an associated phenotype ensemble (here represented by a pie chart of colours), and edges (grey lines) indicate that two genotypes are only a single point mutation apart. **C)** Once the GP map is given, it can be characterised using the definitions in Table 1.

Such analyses have typically worked with deterministic GP maps, where each genotype corresponds to a single, categorical phenotype, see for example [4]. However, deterministic GP maps ignore one central aspect of biological systems: a genotype can produce several phenotypes [5,6]. For example, an RNA sequence does not simply fold into a single structure [7], but is better described by an ensemble, where several structures *p* are present in different ratios *P*(*p*|*g*). Similarly, proteins can have multiple folds [8], self-assembling building blocks can assemble into multiple structures [9], and translation errors generate a specific distribution of protein sequences from an RNA sequence [10]. These examples are captured by GP maps that are *non-deterministic* (ND), meaning that each genotype maps to a probability distribution of phenotypes (see Fig 1A and 1B). Such ND GP maps [1], also known as *plastic* [11]/*many-to-many* [3]/*probabilistic* [2] maps, are fundamentally different from those without ND: Without ND, every genotype corresponds to a single phenotype and thus, every mutation can fall into two classes, either fully phenotype-preserving or generating a new phenotype in a single mutation. With ND, each genotype maps to a phenotype ensemble and mutations can shift the ensemble probabilities by arbitrary amounts. Thus, ND GP maps are more complex.

The higher complexity of ND GP maps is also reflected in the size of these maps: a GP map with alphabet size *K* and sequence length *L* already has a large number of *K*^*L*^ genotypes. Instead of mapping each of these genotypes to a single phenotype, ND GP maps translate each genotype to an *ensemble* over phenotypes, i.e., a set of phenotypes and associated probabilities. Given this inherent complexity, research on ND GP maps would benefit from simple toy models, which replicate shared features of biophysical ND GP maps, but can be modified in a controlled way and are computationally, mathematically and conceptually more tractable. Such toy models can then be used in simulations to investigate how the shared features of ND GP maps shape evolutionary processes. Identifying a suitable simple model is the main goal of this paper.

**Fig 2:**
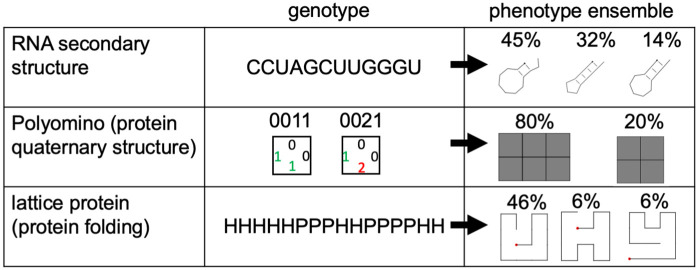
Non-determinism (ND) is present in well-studied biophysical GP maps: Three well-studied biophysical GP map models are non-deterministic, and only simplified treatments fit the deterministic GP map framework.RNA secondary structure [12,13]: each sequence can fold into multiple structures *p*, each with a Boltzmann weight *P*(*p*|*g*) [14]. Polyomino model (simplified model of protein quaternary structure self-assembly): the genotype is a sequence of integers and defines the binding possibilities of a set of self-assembling 2D tiles (in the pictured example, 1 and 2 bind) [15]. Stochastic self-assembly can give multiple shapes, each with a probability *P*(*p*|*g*) [9]. Lattice protein model (simplified model of protein tertiary structure) [16]: a genotype consists of hydrophobic (H) and polar (P) amino acid residues and can fold into multiple structures *p* [17], each with a Boltzmann weight *P*(*p*|*g*).

A suitable simple model should reproduce shared features of biophysical ND GP maps despite its simplicity. For these tests, this paper relies on recent definitions for quantitative features like phenotypic frequencies and evolvabilities in ND GP maps [1,2] - these are summarised in Table 1 and applied to an example in Fig 1C. To confirm, which shared features the simple model should reproduce, these definitions are also applied to the following three biophysical ND GP maps (see Fig 2): the hydrophobic-polar (HP) lattice model [16], a simple model of protein tertiary structures, the tile-based Polyomino self-assembly model [15] mimicking protein quaternary structure and the RNA secondary structure model [13]. All three are classic models in the field [4], but are usually treated in a simplified way that neglects their non-deterministic nature. Out of these three, only the RNA map has been analysed in its full non-deterministic form with the framework in Table 1 [1,2], and is included for completeness. The non-deterministic version of the Polyomino map has been analysed with an alternative framework, where ensemble probabilities first need to be binarised [9].

**Table 1:** Definitions of quantitative ND GP map properties (refs [1,2] with simplified notation): The GP map is needed as an input (for example from a biophysical model), encoded as follows: *P*(*p*|*g*) denotes the ensemble probability of phenotype *p* for genotype *g*. Note that the definitions for frequency and robustness are normalised between zero and one through the constants *K*^*L*^ (number of genotypes in the map) and (*K* − 1)*L* (number of genotypes in a mutational neighbourhood), to ensure comparability across maps and allow an intuitive interpretation as probabilities. Evolvability on the other hand is non-normalised and gives the *number* of distinct accessible alternative phenotypes.

name & description for GP maps *without* ND	analogous definition for ND GP maps
**phenotypic frequency**: fraction of genotypes generating *p*, equivalent to the mean probability of finding *p* in a randomly sampled genotype	${\tilde{f}}_{p}=\frac{1}{K^{L}}\sum_{g}P(p|g)$
**genotypic robustness**: probability that a random mutation on genotype *g* is phenotype-conserving	${\tilde{\rho }}_{g}=\frac{1}{(K-1)L}\sum_{p}P(p|g)\sum_{g^{\prime }\in {𝒩}_{g}}P(p|g^{\prime })$
**phenotypic robustness**: probability that a random mutation is phenotype-conserving, given that the initial phenotype was *p*	${\tilde{\rho }}_{p}=\frac{1}{K^{L}\cdot {\tilde{f}}_{p}\cdot (K-1)L}\sum_{g}P(p|g)\sum_{g^{\prime }\in {𝒩}_{g}}P(p|g^{\prime })$
**genotypic evolvability**: number of phenotypes reachable after a single mutation from genotype *g*	${\tilde{\epsilon }}_{g}=\sum_{p}P(p|g)\sum_{p^{\prime }\neq p}(1-\Pi_{g^{\prime }\in {𝒩}_{g}}(1-P(p^{\prime }|g^{\prime })))$
**phenotypic evolvability**: number of phenotypes reachable after single mutation from any genotype with phenotype *p*	${\tilde{\epsilon }}_{p}=\sum_{p^{\prime }\neq p}(1-\Pi_{g}\Pi_{g^{\prime }\in {𝒩}_{g}}(1-P(p^{\prime }|g^{\prime })P(p|g)))$

*K* is the alphabet size and *L* the sequence length of the genotypes. $\sum_{p}$, $\sum_{g}$ and $\sum_{g^{\prime }\in {𝒩}_{g}}$ are sums over phenotypes, over genotypes and over the mutational neighbourhood of *g* respectively. $\Pi_{g^{\prime }\in {𝒩}_{g}}$ and $\Pi_{g}$ are products over all the genotypes in the mutational neighbourhood of *g*, and over all genotypes.

Following the existing ND GP map analysis of the RNA map [1,2], the side-by-side comparison will be guided by three features that are well-studied in deterministic GP maps [4], for which they can be summarised as follows:

1. The overall prevalence of a phenotype across all genotypes, i.e., the phenotypic frequency, differs from phenotype to phenotype, often by several orders of magnitudes (*phenotypic bias*) [13,15,16].
2. The robustness of a phenotype is higher than its frequency, which means that the probability that genotype *g* maps to phenotype *p* tends to be higher if *g* has *p* among its mutational neighbours (*genetic correlations*) [18].
3. A *genotype* that is robust, i.e., for which a high fraction of mutations are phenotype-preserving, must have low evolvability, i.e., only have a small number of distinct phenotypes accessible through mutations [19]. However, a high-robustness *phenotype* can be produced by many genotypes, with a high combined number of neighbours and thus high evolvability [19].

These three features, their possible roots [20] and evolutionary implications [21–24], are well-studied in deterministic GP maps, but research is needed for more complex and qualitatively different ND GP maps. As a basis for such investigations, the current paper establishes a simple, tuneable model for these ND GP maps.

The paper is structured as follows: first, the simple, synthetic model is defined. Then the features of this synthetic ND GP map are analysed side-by-side with those of the three biophysical ND GP map models. I find that the ND GP maps of both the biophysical and the synthetic models share the three features reviewed above (with some exceptions in the Polyomino model), except the phenotypic robustness-evolvability relationship, which is non-negative but not always clearly positive. These parallels suggest that the synthetic model can serve as a simpler model to facilitate future work on ND GP maps. Finally, I show that the shared features also emerge in several synthetic models built with alternative modelling choices, illustrating that these shared characteristics easily arise from few ingredients and are not special features of biophysical models.

## 2. Results

### 2.1. Defining a simple synthetic ND GP map

To produce an ND GP map, the synthetic model needs to map a genotype $\vec{g}$, i.e., a sequence of characters from a fixed alphabet, to an ensemble of phenotypes, given by a valid probability distribution (i.e., non-negative ensemble frequencies summing to one). This is achieved by the following Boltzmann-ensemble-inspired function:


$$\begin{array}{c} P(p|\vec{g})\propto e^{-G_{p,\vec{g}}/T} \end{array}$$ (1)

Normalising over all *n*_*p*_ phenotypes *q* gives:


$$\begin{array}{c} P(p|\vec{g})=\frac{e^{-G_{p,\vec{g}}/T}}{\sum_{q}e^{-G_{q,\vec{g}}/T}} \end{array}$$ (2)

Here, *T* is analogous to the temperature in a Boltzmann ensemble. Thus, varying *T* from T→0 to T→∞ takes the map from the deterministic limit, which is dominated by the lowest-*G* phenotype per genotype, to the extremely non-deterministic limit, in which all phenotypes have probabilities $P(p|g)=1/n_{p}$ for any genotype *g*. In this limit the map becomes an extreme case of an existing null model, which is defined to have a genotype-independent ensemble with *P*(*p*|*g*) only depending on *p* [2].

$G_{p,\vec{g}}$ is analogous to the free energy and needs to be a scalar depending on both genotype and phenotype. For simplicity, the model will take genotypes from an alphabet of ‘1’ and ‘-1’, which can be represented as an *L*-dimensional vector $\vec{g}$. Then, a scalar quantity can be obtained from $\vec{g}$ using a linear, additive function, which can be written as a dot product:


$$\begin{array}{c} G_{p,\vec{g}}=-\vec{g}\cdot {\vec{v}}_{p}=-\sum_{i}{\vec{g}}_{i}({\vec{v}}_{p}{)}_{i} \end{array}$$ (3)

Here, the phenotype-dependent, *L*-dimensional vector ${\vec{v}}_{p}$ contains the parameters of the linear function. It only needs to be initialised once for each phenotype *p* and is then used for all 2^*L*^ genotypes in the map. To avoid setting additional parameters, each element of ${\vec{v}}_{p}$ is drawn from a normal distribution (with mean and standard deviation set to one). These steps define a synthetic ND GP map with only three parameters to choose: sequence length *L*, number of phenotypes *n*_*p*_ and stochasticity *T*.

This synthetic GP map builds on existing models, which first compute scalars that are linear in the genotype, and then combine these scalars with non-linear functions [25]. Despite the simplicity of such models, with only *O*(*L*) parameters for *K*^*L*^ sequences, such models have been successful at fitting and extrapolating empirical datasets [26–30], sometimes in an extended form that permits second-order terms in constructing the scalars *G* [31,32]. Such models have also served in bottom-up computational models for different purposes, such as investigating mutational bottlenecks [33] and the evolution of interdependent traits like protein folding and binding [34,35]. The synthetic GP map in the current paper closely resembles these bottom-up computational models, with their randomly sampled coefficients and Boltzmann-like nonlinearity [33–35] and thus builds on the success of such models. However, the synthetic ND GP map differs from typical existing setups in having not only a few, but tens to hundreds of phenotypes. This high number of phenotypes is needed to compare the model directly to our biophysical GP map models and thus use this successful class of models for a new question: whether it reproduces shared GP map properties like phenotypic bias and genetic correlations, and what parameters are required to do so.

The following sections will go through the GP map features reviewed in the introduction, and analyse the three biophysical and the synthetic ND GP maps side-by-side, including several choices of *n*_*p*_ and T, for a fixed sequence length *L* = 15. To avoid numerical artefacts and for biological realism, $P(p|g)<10^{-4}$ will be treated as *P*(*p*|*g*) = 0.

### 2.2. Phenotypic bias is present in all biophysical maps, and reproduced by the synthetic model

**Fig 3:**
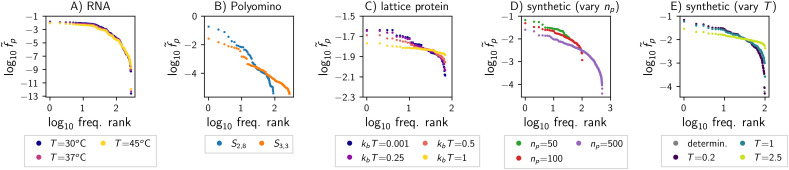
Phenotypic bias in the biophysical GP maps and the synthetic model: Each plot shows the phenotypic frequency ${\tilde{f}}_{p}$ against the frequency rank, i.e., the position of *p* in a list sorted by frequency (shown for all phenotypes appearing in each map, i.e., those with ${\tilde{f}}_{p}>0$). In each map, phenotypic frequencies differ from phenotype to phenotype, and thus all maps show phenotypic bias. The following maps are shown: **A)** RNA secondary structure for different folding temperatures (this map had been analysed [1,2] and is included for completeness); **B)** Polyomino self-assembly model for two parameters (two-tile *S*_2, 8_ and three-tile *S*_3, 3_); **C)** lattice protein model for different folding temperatures; **D)** synthetic model with different numbers of phenotypes *n*_*p*_ (to maintain a comparable level of non-determinism, *T* is set to the genotypic average of the $G_{p,\vec{g}}$-difference between the lowest two phenotypes); **E)** synthetic model with different stochasticity *T* for fixed *n*_*p*_ = 100 (including the deterministic limit T→0, where each genotype maps to the lowest-*G* structure).

Let us start with the phenotypic frequency ${\tilde{f}}_{p}$, i.e., the mean ensemble frequency of a phenotype *p* across all genotypes and thus its overall abundance. For each GP map, Fig 3 plots this frequency of a phenotype *p* against its rank in the corresponding GP map, i.e., against the position of *p* in a list sorted by frequency. In all three biophysical GP maps as well as the synthetic model, we find phenotypic bias, in agreement with previous work for RNA [1] and lattice proteins [36]: different phenotypes in a single GP map have different ${\tilde{f}}_{p}$.

**Fig 4:**
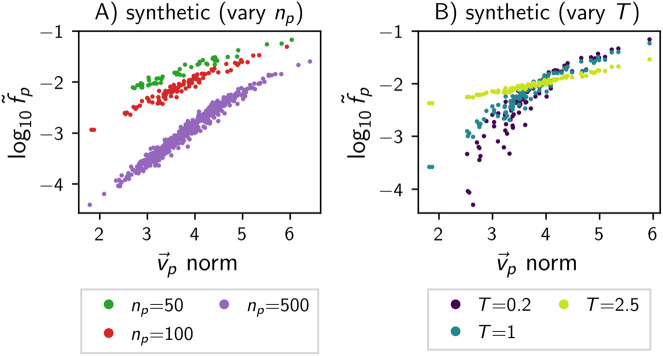
In the synthetic model, random differences in the parameter vectors are linked to phenotypic frequency differences: For each phenotype appearing in the map (i.e., ${\tilde{f}}_{p}>0$), the phenotypic frequency ${\tilde{f}}_{p}$ is plotted against the vector norm of the phenotype’s parameter vector ${\vec{v}}_{p}$. The models are the same as in Fig 3D and 3E (see subplot titles).

Focusing first on the biophysical GP maps, we see that the RNA and Polyomino map both have phenotypic frequency differences spanning several orders of magnitude. In contrast, in the lattice protein model, the relative differences in phenotypic frequencies are smaller. Turning to the synthetic map, we see that phenotypic frequencies also span several orders of magnitude and thus the model can produce strong phenotypic bias. This bias must emerge from phenotype-dependent aspects of the model and thus from random differences in the parameter vectors ${\vec{v}}_{p}$. Concretely, let us consider the following hypothesis: a vector with larger absolute elements can give more favourable values of $G_{p,\vec{g}}$, which are present in many genotypes and amplified by the non-linear function in eq. 2, leading to the observed frequency differences of several orders of magnitude. To test this hypothesis, the vector norm of $\vec{v_{p}}$ is plotted against the frequency ${\tilde{f}}_{p}$ of each phenotype *p* in Fig 4, giving a positive trend as hypothesised. This argument is reminiscent of research on the deterministic GP maps of RNA and lattice proteins [37,38], in that phenotypes with low *G* values in a higher number of genotypes have higher phenotypic frequencies.

Comparing synthetic maps constructed with different parameters, we find phenotypic bias to weaken with increasing stochasticity *T*. A higher stochasticity *T* reduces the impact of phenotypic differences in $G_{p,\vec{g}}$ on ensemble probabilities for each individual genotype (see eq. 2). Thus, phenotypic differences will also be less pronounced when summed over all genotypes to obtain phenotypic frequencies ${\tilde{f}}_{p}$. In the extreme case T→∞, phenotypic differences vanish entirely: any genotype *g* maps to any phenotype *p* with the same ensemble probability $P(p|g)=1/n_{p}$, giving ${\tilde{f}}_{p}=\frac{1}{K^{L}}\sum_{g}\frac{1}{n_{p}}=\frac{1}{n_{p}}$ for all phenotypes and thus no phenotypic bias. Thus, the weak phenotypic bias in the lattice protein model is reminiscent of the high-stochasticity limit in the synthetic ND GP map. This parallel may reflect the high stochasticity in the lattice protein model: ≈62% of lattice protein genotypes have more than one minimum-energy structure and in these genotypes even the highest ensemble frequency cannot reach 0.5.

### 2.3. Genetic correlations in most biophysical ND GP maps and the synthetic model

**Fig 5:**
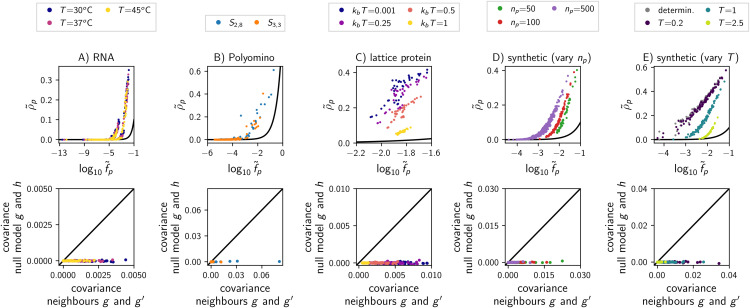
Genetic correlations in biophysical GP maps and the synthetic model: The GP maps in each column are the same as in Fig 3 (see subplot titles).(first row) For each phenotype appearing in the map (i.e., ${\tilde{f}}_{p}>0$), phenotypic robustness ${\tilde{\rho }}_{p}$ is plotted against the log of the phenotypic frequency $\log_{10}{\tilde{f}}_{p}$ (with ${\tilde{f}}_{p}={\tilde{\rho }}_{p}$ indicated by a black line). ${\tilde{\rho }}_{p}>{\tilde{f}}_{p}$ implies genetic correlations. (second row) Covariance analysis to detect genetic correlations (only ND maps, not for deterministic limit): 10^4^ pairs of mutational neighbours, *g*/$g^{\prime }$, were generated at random. *P*(*p*|*g*) and $P(p|g^{\prime })$ were recorded for each pair and each phenotype. Then, for each phenotype *p*, the covariance was computed between the *P*(*p*|*g*) data and the corresponding $P(p|g^{\prime })$ data (x-axis). The zero-correlation null model (y-axis) was computed in the same way, except that a random genotype *h* replaced the mutational neighbour $g^{\prime }$. Here, genetic correlations are reflected in covariances that are positive and exceed the null model.

Next, let us focus on genetic correlations, i.e., whether the robustness ${\tilde{\rho }}_{p}$ of a phenotype is higher than its phenotypic frequency ${\tilde{f}}_{p}$. The first row of Fig 5 shows that such genetic correlations exist across most of the biophysical and synthetic GP maps, as previously demonstrated for RNA [1,2].

However, in the Polyomino *S*_3, 3_ model, there are exceptions where the condition for genetic correlations, ${\tilde{\rho }}_{p}>{\tilde{f}}_{p}$, is not met: while 74 out of 277 phenotypes satisfy ${\tilde{\rho }}_{p}>{\tilde{f}}_{p}$, the remaining phenotypes have low non-zero frequencies and zero robustness. Thus, for the Polyomino *S*_3, 3_ map, the verdict on genetic correlation depends on the perspective: the phenotypes displaying genetic correlations make up 94% of the combined phenotypic frequencies of all phenotypes, and thus dominate on the genotypic level, but they constitute a minority on the phenotypic level. The interpretation of zero robustness values is further complicated by numerical inaccuracies: Zero robustness values imply that a genotype with that phenotype in its ensemble has no mutational neighbours with that phenotype in its ensemble. This calculation may miss cases with 0 < *P*(*p*|*g*) < 0.01 in the neighbouring genotypes, which are not reported in the Polyomino ensembles due to potential samplin*g* errors (see methods 4.3).

When comparing the synthetic GP map with different stochasticity parameters *T*, we find that a higher stochasticity tends to weaken genetic correlations, in agreement with the trends previously found in the highly stochastic limit in the RNA, spin-glass and quantum circuit models [2]. This is best understood in the extreme limit T→∞, where $P(p|g)=1/n_{p}$ for all phenotypes and genotypes, and thus ${\tilde{f}}_{p}={\tilde{\rho }}_{p}=1/n_{p}$ for all phenotypes. Thus, in the T→∞ limit, there is no local, genotype-dependent structure and no genetic correlations.

The presence of genetic correlations, as well as the existence of outliers in the Polyomino model, are further supported by covariance analyses: when ${\tilde{\rho }}_{p}>{\tilde{f}}_{p}$, we expect a positive covariance when plotting *P*(*p*|*g*) against $P(p|g^{\prime })$ for pairs of neighbouring genotypes *g* and $g^{\prime }$ (see section A.I in S1 Text for a derivation of this equivalence). As expected, covariances between mutational neighbours are mostly positive and mostly exceed the covariances between random, non-neighbouring genotypes across our GP maps (second row of Fig 5). In line with the previous analysis, exceptions are most prominent in the Polyomino *S*_3, 3_ model, where they constitute the majority on the phenotypic level.

**Fig 6:**
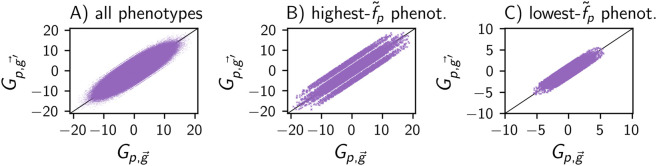
In the synthetic model, mutational neighbours *g* and $g^{\prime }$ have correlated $G_{p,\vec{g}}$ and $G_{p,\vec{g^{\prime }}}$: (A) $G_{p,\vec{g^{\prime }}}$ is plotted against $G_{p,\vec{g}}$ for all phenotypes *p* in 10^4^ pairs of randomly generated mutational neighbours, *g*/$g^{\prime }$. **(B)** Same plot for one phenotype, the highest-${\tilde{f}}_{p}$ phenotype, to control for phenotypic differences in $G_{p,\vec{g}}$. **(C)** Same for lowest-${\tilde{f}}_{p}$ phenotype. Since $G_{p,\vec{g}}$ does not depend on *n*_*p*_ or *T*, data is simply shown for the *n*_*p*_ = 500 model from Fig 3D.

This interpretation of genetic correlations, as a positive covariance between neighbouring ensembles, allows us to hypothesise their origin in the synthetic model: A single mutation only changes a single site of the genotype and thus a single summand in the dot product of eq. 3. Thus, if phenotype *p* has low $G_{p,\vec{g}}$ for genotype $\vec{g}$, *p* also tends to have low $G_{p,\vec{g^{\prime }}}$ for genotypes $\vec{g^{\prime }}$ that are one point mutation away from $\vec{g}$, as seen in Fig 6. Since $G_{p,\vec{g}}$ influences the ensemble probability via eq. 2 (see section B in S1 Text), this implies correlations in ensemble probabilities and thus genetic correlations.

### 2.4. Genotypic robustness and evolvability follow a trade-off

**Fig 7:**
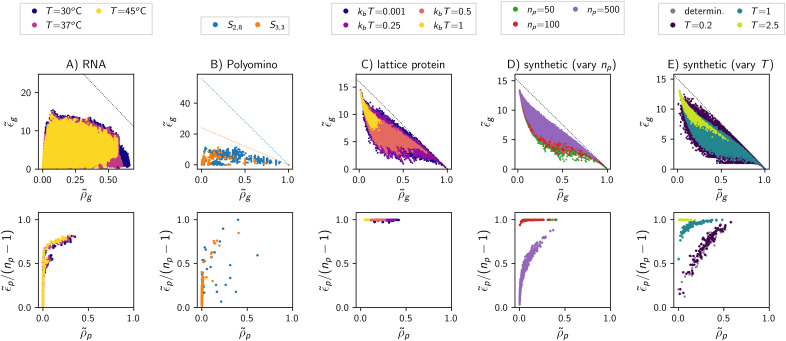
Evolvability and robustness satisfy a trade-off at the genotypic level, but a non-negative relationship on the phenotypic level: (first row) Genotypic evolvability ${\tilde{\epsilon }}_{g}$ is plotted against genotypic robustness ${\tilde{\rho }}_{g}$ (deterministic limit of synthetic model not included to reduce overlapping data). There are no high-evolvability-high-robustness genotypes, consistent with the upper bound ${\tilde{\epsilon }}_{g}\leq (K-1)L(1-{\tilde{\rho }}_{g})$ (dashed lines; the data falls below the bound when accounting for numeric errors of up to 10^−4^). Since the two versions of the Polyomino GP map have different values of *K* and *L*, each has their own upper bound; all other GP maps only have a single upper bound shown in black. (second row) Phenotypic evolvability ${\tilde{\epsilon }}_{p}$ is normalised relative to its maximum *n*_*p*_ − 1 (where *n*_*p*_ is the number of phenotypes with non-zero frequencies in the map). This normalised phenotypic evolvability is plotted against phenotypic robustness ${\tilde{\rho }}_{p}$, showing a (weak) positive trend in some maps (esp. RNA and synthetic map with high *n*_*p*_ and low *T*) and a saturation at the maximum in others (esp. lattice protein and synthetic map with low *n*_*p*_ or high *T*). The models are the same as in Fig 3 (see column titles).

Let us now turn to the relationship between the robustness ${\tilde{\rho }}_{g}$ and evolvability ${\tilde{\epsilon }}_{g}$ of individual *genotypes*. In the deterministic case, genotypes have a limited number of mutational neighbours and thus cannot combine high robustness with high evolvability, giving $(1-\rho_{g})(K-1)L\geq \epsilon_{g}$ [19]. This trade-off generalises to the ND case (section A.II in S1 Text) and is consistent with the data for the three biophysical maps as well as for the synthetic model (first row of Fig 7), in agreement with previous analyses in the RNA case [1].

Beyond checking whether the trade-off is satisfied, let us focus on the genotypic robustness and evolvability data in more detail: some maps, such as the low-T versions of the lattice protein and synthetic model, have a range of robustness and evolvability values, and different combinations of robustness and evolvabilities within the range permitted by the trade-off. However, the high-*T* versions of the synthetic model and the lattice protein model only contain low-robustness-high-evolvability genotypes. This can be understood by considering the synthetic model in the extreme limit T→∞ with $P(p|g)=1/n_{p}$ for all phenotypes *p* and genotypes *g*, giving:


$$\begin{array}{c} {\tilde{\rho }}_{p}=\frac{1}{K^{L}\cdot {\tilde{f}}_{p}\cdot (K-1)L}\sum_{g}\frac{1}{n_{p}}\sum_{g^{\prime }\in {𝒩}_{g}}\frac{1}{n_{p}}=\frac{1}{n_{p}} \end{array}$$

$$\begin{array}{c} {\tilde{\epsilon }}_{g}=\sum_{p}\frac{1}{n_{p}}\sum_{p^{\prime }\neq p}(1-\Pi_{g^{\prime }\in {𝒩}_{g}}(1-\frac{1}{n_{p}})) \end{array}$$

$$\begin{array}{c} =(n_{p}-1)(1-(1-\frac{1}{n_{p}}{)}^{(K-1)L})\approx (n_{p}-1) \end{array}$$

Thus, in the high-*T* limit, all genotypes in the synthetic map have high evolvability close to the maximum of *n*_*p*_ − 1 and low robustness.

### 2.5. Phenotypic robustness and evolvability follow a non-negative trend

Despite the trade-off between *genotypic* evolvability and robustness, the *phenotypic* analogues can be positively correlated. This is simplest to understand for deterministic GP maps: more robust phenotypes have higher phenotypic frequencies, and thus more mutational neighbours overall, giving them the potential for higher evolvability [19]. This trend can continue until the maximum possible evolvability value, which is given by the total number of alternative phenotypes *n*_*p*_ − 1.

When plotting phenotypic evolvability against robustness in the biophysical ND GP maps as well as the synthetic model, the results are mixed (second row of Fig 7): In the RNA map as well as in the synthetic map for a low stochasticity *T*, there is a clear non-linear positive trend, in agreement with previous work on the RNA model [1].

In the Polyomino map, the trend in the robustness-evolvability relationship is not as simple: while the three-tile map *S*_3, 3_ suggests a non-linear positive trend, the trend in the two-tile system *S*_2, 8_ is less clear. This may be an artefact of the relatively small system size of two or three tiles, chosen for reasons of computational feasibility.

In the lattice protein model, all phenotypes have evolvabilities within 4% of the maximum, and thus there is no relevant trend. This close-to-maximum evolvability in the lattice protein model is also seen in the synthetic model in two parameter regimes: a low number of phenotypes *n*_*p*_ or high stochasticity *T*. For GP maps with a low number of phenotypes *n*_*p*_, the maximum possible evolvability value ${\tilde{\epsilon }}_{p}=n_{p}-1$ is low and therefore reached by a higher fraction of phenotypes. In GP maps with high stochasticity *T*, the phenotypic diversity in any mutational neighbourhood is high, demonstrated by the high *genotypic* evolvabilities, leading to high phenotypic evolvability values. In the extreme limit T→∞, all phenotypes have evolvabilities close to the maximum of *n*_*p*_ − 1:


$$\begin{array}{c} {\tilde{\epsilon }}_{p}=\sum_{p^{\prime }\neq p}(1-\Pi_{g}\Pi_{g^{\prime }\in {𝒩}_{g}}(1-\frac{1}{n_{p}}\frac{1}{n_{p}})) \end{array}$$

$$\begin{array}{c} =(n_{p}-1)(1-(1-\frac{1}{n_{p}^{2}}{)}^{K^{L}(K-1)L})\approx (n_{p}-1) \end{array}$$

Thus, the close-to-maximum phenotypic evolvability is a second parallel between the high-*T* limit of the synthetic model and the lattice protein model, besides their weak phenotypic bias, suggesting that the high stochasticity of the lattice protein model may be one reason for its role as an outlier.

### 2.6. A wide range of modelling choices in the synthetic ND GP map reproduce the shared features

Our analyses have shown that the simple synthetic model is able to reproduce central features of the biophysical models: strong phenotypic bias, genetic correlations and a robustness-evolvability relationship that satisfies a trade-off on the genotypic level, but is non-negative on the phenotypic level. These features are present for a range of parameter values of *n*_*p*_ and *T*, but it is not clear, whether they depend strongly on the other choices made when designing the synthetic model. Thus, I considered five alternative functional forms that could replace the Boltzmann-like function in the synthetic model definition (eq. 1): shifted linear, inverse-squared, Gaussian, ReLu and Softplus functions. All five functional forms were implemented in a way that ensured that all probabilities are non-negative, that higher-$G_{p,\vec{g}}$ phenotypes have lower ensemble probabilities and that ensemble frequency differences become weaker with increasing *T*, with $P(p|\vec{g})\to 1/n_{p}$ in the limit T→∞. These conditions led to the functional forms in Table 2. Note that the ‘linear’ functional form has a shifted linear numerator $P(p|\vec{g})\propto G_{\text{max},\vec{g}}-G_{p,\vec{g}}+T$, but will be non-linear when the probabilities are normalised to give $P(p|g)=(G_{\text{max},\vec{g}}-G_{p,\vec{g}}+T)/(\sum_{q}(G_{\text{max},\vec{g}}-G_{q,\vec{g}}+T))$.

**Table 2:** Functional forms as alternatives to eq. 1.

label in Fig 8	functional form
linear	$P(p|\vec{g})\propto G_{\text{max},\vec{g}}-G_{p,\vec{g}}+T$
inverse-squared	$P(p|\vec{g})\propto (G_{p,\vec{g}}-G_{\text{min},\vec{g}}+T{)}^{-2}$
Gaussian	$P(p|\vec{g})\propto \exp(-(G_{p,\vec{g}}-G_{\text{min},\vec{g}}{)}^{2}/T)$
ReLu	$P(p|\vec{g})\propto \text{max}(T-G_{p,\vec{g}},0)$
Softplus	$P(p|\vec{g})\propto \ln(1+e^{T-G_{p,\vec{g}}})$

**Fig 8:**
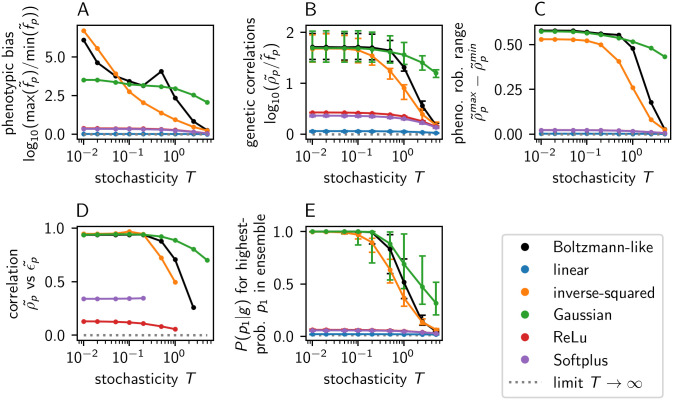
ND GP map analysis for synthetic models built with alternative functional forms (*n*_*p*_ = 100): **(A)** Log-ratio of the highest to lowest phenotypic frequencies as a proxy for phenotypic bias. **(B)** Log-ratio of phenotypic robustness to frequency as a proxy for genetic correlations (median and quartiles over all phenotypes with $\tilde{f_{p}}>0$ and $\tilde{\rho_{p}}>0$). **(C)** Difference between minimum and maximum phenotypic robustness ${\tilde{\rho }}_{p}$ in the map. **(D)** Pearson correlation coefficient between robustness and evolvability on the phenotypic level (no value is computed if maximum and minimum evolvability values differ by less than 10^−6^). **(E)** Typical $P(p|\vec{g})$ of highest-ranking phenotype *p* in the ensemble of a given genotype $\vec{g}$ (median and quartiles over all genotypes shown). The labels for the alternative functional forms are given in the legend and defined in Table 2. The Boltzmann-like form used previously (eq. 1) is shown in black for reference.

Equipped with the alternative functional forms in Table 2, I repeated central aspects of the ND GP map analysis. I find that the results are qualitatively unchanged if the Boltzmann-like functional form is exchanged for the inverse-squared or Gaussian functional forms (Fig 8). For a range of parameter values of *T*, there is strong phenotypic bias: We have $\log_{10}(\text{max}({\tilde{f}}_{p})/\text{min}({\tilde{f}}_{p}))>1$ and the highest and lowest non-zero phenotypic frequencies differ by at least an order of magnitude (Fig 8A). Secondly, these three maps display clear genetic correlations, with typical phenotypes satisfying $\log_{10}(\tilde{\rho_{p}}/{\tilde{f}}_{p})>1$ and thus having a ten-fold higher robustness $\tilde{\rho_{p}}$ than phenotypic frequency $\tilde{f_{p}}$ (Fig 8B). Thirdly, in these maps, phenotypic robustness $\tilde{\rho_{p}}$ differs from phenotype to phenotype, with the minimum and maximum robustness differing by > 0.5 (Fig 8C). These robustness differences are positively correlated with phenotypic evolvability $\tilde{\epsilon_{p}}$ (Fig 8D). As *T* increases and individual ensembles become less biased towards a small number of low-*G* phenotypes (Fig 8E), the features become closer to the T→∞ limit, as expected. However, the convergence to the T→∞ limit is non-monotonic in Fig 8A, partly because $\log_{10}(\text{max}({\tilde{f}}_{p})/\text{min}({\tilde{f}}_{p}))$ is especially sensitive to the lowest-frequency phenotypes, and these can be absent from the map for some values of *T*, if they do not have $P(p|g)>10^{-4}$ for at least one genotype.

In contrast, the ND GP maps built with the linear, ReLu and Softplus functions show much weaker phenotypic bias, weaker genetic correlations, a smaller range of phenotypic robustness values and a weaker or absent trend in the phenotypic robustness-evolvability relationship (Fig 8). This can be explained by considering the ensemble of a typical genotype (Fig 8E): the linear, ReLu and Softplus functions do not produce strong bias even within a single genotype’s ensemble, and the highest-$P(p|\vec{g})$ value for a given genotype $\vec{g}$ falls much closer to an unbiased ensemble with $P(p|\vec{g})=1/n_{p}$ than in the other functional forms. This resembles the high-*T* limit in the other maps, for which we previously found weak phenotypic bias, weak genetic correlations and small robustness differences from phenotype to phenotype in the‌‌ map.

This resemblance to the high-*T* limit is easy to understand for the linear function, which lacks a non-linearity biasing individual ensembles towards low-$G_{p,\vec{g}}$ phenotypes. A similar argument applies to the ReLu function and to the closely related Softplus function: the ReLu has a linear segment for $G_{q,\vec{g}}\leq T$. A high number of $G_{q,\vec{g}}$ values fall into this linear segment since $G_{q,\vec{g}}$ is equally likely to be negative as positive (by symmetry). The remaining $G_{q,\vec{g}}$ values with $G_{q,\vec{g}}\geq T$ *are* suppressed because the ReLu function is non-linear, with a flat segment for $G_{q,\vec{g}}>T$. This explains why the ReLu function produces more phenotypic bias and genetic correlations than the linear version. This suppression of high-*G* phenotypes could be strengthened if we allowed *T* < 0, thus reducing the number of $G_{q,\vec{g}}$ values falling into the linear segment of the ReLu function. In this case, the ReLu function may generate stronger bias, but would no longer be relevant as a positive-definite function approximating a *linear* trend.

Beyond the functional form, a second modelling choice in the synthetic model is the probability distribution used to initialise the parameter vectors ${\vec{v}}_{p}$. Thus, I repeated the analysis for two further probability distributions: a log-normal distribution as one example of distributions with a more pronounced tail and a uniform distribution as one example of distributions with a fixed maximum value. Moreover, I added a fourth parameter initialisation scheme: after drawing the vectors ${\vec{v}}_{p}$ from a normal distribution, I took the mean vector norm across all parameter vectors and rescaled all vectors to this mean value. This renormalisation removes differences in the vector norm, which Fig 4 showed to be linked to phenotypic bias. As an additional alternative, I initialised the vectors ${\vec{v}}_{p}$ from a discrete ‘binary’ probability distribution, drawing either 0.25 or 0.75 with equal probability. This mimics the lattice protein model, where many genotypes have highly non-deterministic ensembles in the low-*T* limit simply because the discrete energy model produces no differences in $G_{p,g}$ between the lowest-lying structures. Finally, I constructed another version of the ND GP map, where the vectors ${\vec{v}}_{p}$ and an additional phenotype-dependent constant are initialised from a normal distribution, and the constant is then added as an offset to the dot product in eq. 3.

**Fig 9:**
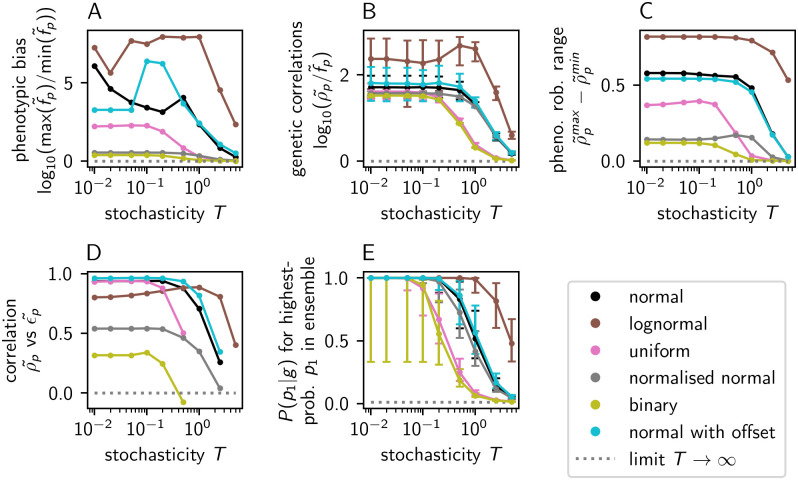
ND GP map analysis for synthetic models built with alternative parameter initialisations: The analysis in (A -E) proceeds as in Fig 8. The initialisation used previously, drawing ${\vec{v}}_{p}$ elements from a normal distribution, is shown in black for reference. The alternatives are: (1) The ${\vec{v}}_{p}$ elements are sampled from a log-normal distribution with μ=0 and σ=1 in the underlying normal distribution. (2) The ${\vec{v}}_{p}$ elements are sampled from a uniform distribution between 0 and 1. (3) The ‘normalised normal’ initialisation keeps the vector norm of ${\vec{v}}_{p}$ fixed: it starts with an initialisation from a normal distribution; then the mean vector norm across all parameter vectors is computed and each vector ${\vec{v}}_{p}$ is rescaled to this mean. (4) The ‘binary’ parameter initialisation, inspired by the discreteness of the lattice protein model, draws ${\vec{v}}_{p}$ elements from two alternatives, 0.25 and 0.75, with equal probability. (5) The ‘normal with offset’ parameter initialisation is based on a normal distribution, and draws one additional phenotype-dependent constant besides ${\vec{v}}_{p}$, which is added to the linear eq. 3 as a constant offset. Note that the absolute value of *T* may have different implications for different parameter initialisations since the *ratio* of *G* to *T* enters the ensemble calculations, and *G* is shaped by the parameter vectors ${\vec{v}}_{p}$.

Analysing the ND GP maps generated by these different parameter vectors ${\vec{v}}_{p}$, we find that the qualitative conclusions are robust to opting for a uniform or log-normal distributions in the ${\vec{v}}_{p}$ initialisation, as well as to including the additional constant offset in eq. 3: synthetic models in the low-*T* limit have strong phenotypic bias (Fig 9A), strong genetic correlations (Fig 9B), a range of at least 0.3 in their phenotypic robustness (Fig 9C) and a positive phenotypic robustness-evolvability relationship (Fig 9D). However, the model with normalised parameter vectors is an exception with much weaker phenotypic bias. This weak bias is consistent with fact that vector norm is closely linked to phenotypic frequency in Fig 4. The remaining bias suggests that further, albeit weaker, sources of bias exist even when the vector norm is kept constant, just like in the deterministic version of the lattice protein GP map, where bias stems from variation in the number of low-free-energy genotypes per phenotype [38], but further sources of frequency differences exist: phenotypes whose low-free-energy genotypes do not coincide with the low-free-energy genotypes of competing phenotypes [39].

Finally, the discrete ‘binary’ distribution resembles the T→∞ limit in some aspects, esp. its weak phenotypic bias and the small range of phenotypic robustness values in the map. This is consistent with the fact that a relevant fraction of genotypes does not have a single phenotype with *P*(*p*|*g*) > 0.5 even in the low-*T* limit. Just as in the lattice protein model, this feature follows from the discrete energy scale: if the lowest-free-ener*g*y-value is shared by two phenotypes, even the most frequent phenotype has P(p|g)→0.5 as T→0. Thus, to generate ND GP maps, where most ensembles are dominated by a single phenotype, one needs to choose not only a functional form to amplify differences in $G_{p,g}$, but also a parameter initialisation that generates such $G_{p,g}$ differences.

A third modification of the synthetic model is possible: changing the alphabet size from a binary alphabet with *K* = 2 to a DNA alphabet with *K* = 4. This change similarly leaves the central shared ND GP map features qualitatively unchanged, see section G in S1 Text.

In conclusion, mechanisms creating sufficient bias, such as non-linearities in combination with a suitable parameter initialisation, are important for building ND GP maps far from the trivial high-*T* limit with $P(p|g)=1/n_{p}$ for all genotypes and phenotypes. Nevertheless, there are several versions of the synthetic model reproducing the shared features of the biophysical maps, suggesting that these features emerge easily from simple models. Of course, these alternative synthetic models may differ in other respects, just like biophysical GP maps show differences in aspects beyond their shared features [40].

## 3. Discussion and conclusions

While many realistic genotype-phenotype (GP) maps are non-deterministic (ND), our understanding of such maps is much less developed than that of simpler GP maps without ND, where every genotype maps to a single categorical phenotype. Here, I build a foundation for a more systematic understanding of ND GP maps by showing that a simple, tuneable non-biological model of an ND GP map reproduces key features of three biophysical ND GP maps (RNA secondary structure, lattice protein model, Polyomino self-assembly model): phenotypic bias, genetic correlations, a tradeoff between *genotypic* robustness and evolvability and a non-negative trend between *phenotypic* robustness and evolvability. These features are present in the synthetic model for a range of parameter values, indicating that they emerge from simple, non-biological models without fine-tuning, and may thus be found more widely.

The synthetic ND GP map combines a linear genotype-dependence (eq. 3) with a non-linear scalar function (eq. 2). Replacing the non-linear function with a normalised and shifted linear function gave only weak phenotypic bias and genetic correlations, which suggests that mechanisms amplifying bias are important. Non-linearities are one such mechanism, but other alternatives may exist.

The combination of additive scalars with a non-linear scalar function builds on a successful series of existing models, both for fitting and extrapolating from data [26–30] and for theoretical analyses [33,34]. Here, such models are built not for a few, but for up to hundreds of phenotypes, in order to use them for a new objective: identifying models of low conceptual and computational complexity that nevertheless reproduce the shared features of ND GP maps.

While ND GP maps are the main goal of the present paper, the synthetic model also defines a deterministic GP map if each genotype is mapped to its highest-*P*(*p*|*g*) phenotype. Thus, it provides an alternative to the Fibonacci model [20,41], an existing minimal model reproducing the shared features of deterministic GP maps. The Fibonacci model has a phenotype-dependent set of *unconstrained* sequence positions, which can mutate without phenotypic effect, as well as phenotype-changing *constrained* positions [20]. This concept of phenotype-dependent *sequence constraints* [20] has been used to approximate more complex GP maps [42–45] and provides one hypothesis for the origin of shared GP map characteristics in the deterministic case (see reviews [3,4]). The synthetic model in the current paper also replicates the shared features of deterministic GP maps, but differs from idealised constraint-based models like the Fibonacci model: the availability of phenotype-conserving, neutral mutations at a given position is sequence-dependent (section F in S1 Text). This sequence-dependence is interesting, since there is some evidence that it also exists in (models of) biological systems: an empirical GP map for transcription factor binding (where the sequence-dependence is seen in the marked variation in genotypic robustness within a phenotype) [46], a Potts-model-fit to the β-lactamase sequence family [47] and empirical fitness effects in orthologous sequences [48]. Future work should investigate these sequence-dependent effects in more detail, including whether they are sufficiently strong for these examples to fall outside the scope of constraint-based models, and such research may take the synthetic model as one example.

The deterministic version of the synthetic GP map may be useful beyond the context of sequence-dependent neutrality: It may also be useful in applications, where several GP maps are needed for testing, since several maps can be built by resampling parameters.

This analysis of robustness and evolvabilities relies on definitions which proceed in close analogy with their established deterministic counterparts and take the full ensemble probabilities into account [1,2]. However, an alternative, threshold-based treatment of ND GP maps has also been proposed, which uses the value of the ensemble frequency only to determine whether a phenotype’s *P*(*p*|*g*) falls above a fixed threshold [9]. These definitions for robustness and evolvabilities could also be applied to the synthetic model if a biologically motivated threshold was given (see section H in S1 Text).

Since the synthetic model replicates key features of biophysical models, it can serve as a tractable model for future work on ND GP maps and their implications for evolutionary processes: The model allows the construction of ND GP maps of arbitrary size, by choosing not only the number of phenotypes *n*_*p*_, but also the number of genotypes and thus ensembles (through the sequence length *L* and alphabet size *K*). In addition, the synthetic model has tuneable parameters *T* and *n*_*p*_, allowing us to generate a family of ND GP maps whose features can be contrasted. Tunable models are valuable for building theory, for example the ‘NK’ and ‘Rough-Mount-Fuji’ models for fitness landscapes [49]. Thus, the synthetic model will help investigate ND GP maps systematically, in particular:

- **Evolutionary simulations:** Quantities like phenotypic frequencies are likely to be relevant in many evolutionary scenarios on ND GP maps, for example under periodically changing selection [50]. However, other quantities like phenotypic evolvability were motivated by deterministic GP maps, where population can drift through the set of phenotypically identical genotypes [19]. In ND GP maps, genotypes dominated by the same phenotype *p* can have different ensemble probabilities *P*(*p*|*g*) and are thus no longer phenotypically identical [11]. Thus, instead of evolvability and other analogues from deterministic maps, new definitions should be established to quantify aspects of ND GP maps relevant for evolutionary predictions under different selective pressures. To disentangle different hypotheses, the tractability of the synthetic model, as well as the ability to generate multiple maps from a single model, is advantageous.
- **Analytic scaling in the large-L limit:** For longer, biologically relevant lengths, it becomes infeasible to exhaustively analyse computational GP maps. Here, the simplicity of the synthetic model may allow us to nevertheless extrapolate some GP map features analytically.
- **Fitting and extrapolating ND GP maps:** For deterministic GP maps, the Fibonacci model [20] established the concept of sequence constraints, which then prompted related constraint-based-models for fitting deterministic GP maps and extrapolating their phenotypic frequencies [42,44]. In a similar way, the synthetic model may provide a foundation for fitting and extrapolating ND GP maps.
- **Developing sampling methods:** Further developments of sampling methods (see [1]) could allow estimates of ND GP map features (${\tilde{f}}_{p}$, ${\tilde{\rho }}_{p}$, ideally even ${\tilde{\epsilon }}_{p}$), from small and local samples. The synthetic model is highly suitable for testing such methods since it is easy to create not just one, but several ND GP maps, by resampling parameters or changing the functional form. Then, these methods could be used to estimate the features of more complex computational models, e.g., gene regulatory networks [51], and from high-throughput experimental data.

These analyses are relevant for a larger class of GP maps beyond ND GP maps: ND GP maps combine discrete and continuos phenotypic information (discrete phenotypes *p* and continuous probabilities *P*(*p*|*g*)), and are therefore conceptually similar to other GP maps with both categorical and quantitative phenotypic information, even if the quantitative components are not probabilities. This would include a wider range of systems: for example, empirical transcription factor binding and RNA-binding protein GP maps [52,53], where each sequence maps to a set of discrete transcription factors/RNA binding proteins, as well as a continuous enrichment score for each transcription factor/RNA binding protein.

## 4. Methods

### 4.1. RNA GP map

For each RNA genotype of length *L* = 12 nucleotides, the Boltzmann ensemble of secondary structures was computed using the ViennaRNA package [12] (version 2.7.0): first, a list was generated with all secondary structures whose base pairs are compatible with the genotype *g*. Then, the free energy $G_{p,g}$ of each structure *p* was calculated with the eval_structure function in ViennaRNA’s Python bindings. Then, the Boltzmann weights follow from the standard relationship [14]:


$$\begin{array}{c} P(p|g)=\frac{\exp(-G_{p,g}/(k_{B}T))}{\sum_{q}\exp(-G_{q,g}/(k_{B}T))} \end{array}$$ (4)

Here, the sum is over all structures *q* that are compatible with *g* (including the unfolded structure with no base pairs).

### 4.2. Lattice protein GP map

In the lattice protein GP map, each structure corresponds to a configuration of a polymer chain on a lattice [16,54], here a chain of length *L* = 16 on a 4 × 4 lattice. First, all configurations need to be generated. To filter for unique configurations, mirror images and rotations are removed [55], but two configurations with reversed chain directions are considered distinct since protein backbones have an inherent directionality.

For a given genotype *g*, made up of hydrophobic (H) and polar (P) residues, the free energy $G_{p,g}$ associated with each configuration *p* is computed based on a given contact potential. Here, Li et al.’s [16] contact energies are used since this HP contact potential gives fewer genotypes with degenerate minimum-energy states than its alternative [55]. Thus, the energies are $c_{HH}=-2.3$ for HH-contacts, $c_{HP}=-1$ for HP contacts and $c_{PP}=0$ for PP contacts [16]. Given $G_{p,g}$, the ensemble frequencies *P*(*p*|*g*) follow from eq. 4. Since the free energy $G_{p,g}$ is dimensionless in this abstract model, $k_{B}T$ is also dimensionless.

### 4.3. Polyomino self-assembly GP map

In the Polyomino model $S_{t,c}$, a length-4*t* genotype is used to label the 4*t* faces of *t* square tiles, where each tile face can take any integer between 0 and *c* − 1 [15]. A face’s integer specifies its binding properties: a face labelled 0 cannot bind, 1 and 2 can bind, 3 with 4 etc. [15].

**Fig 10:**
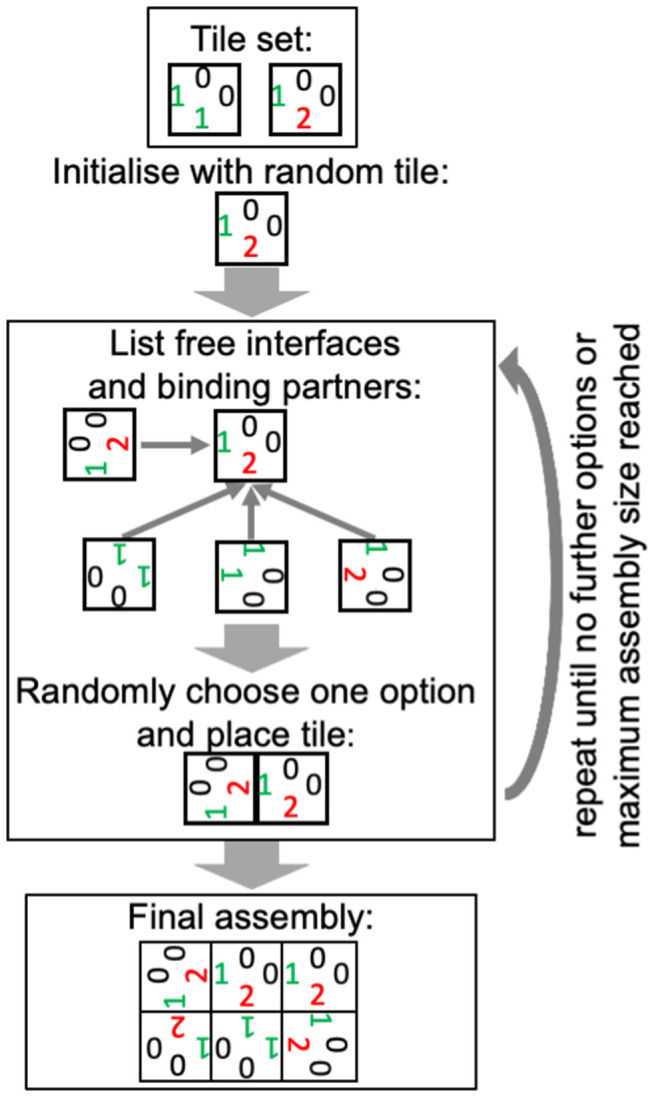
Polyomino self-assembly (following [56]): The orientations of the numbers serve to visualise rotations of the original tiles. Only one interface needs to match for a tile to be added, so not all touching edges in the final assembly have to bind. The maximum assembly size ((4*t*)^2^/2 for *t* tiles) is needed because infinite assemblies are possible.

To go from a genotype - and its encoded set of tiles - to an ensemble of phenotypes, stochastic self-assembly was simulated as shown in the schematic in Fig 10.

Due to the randomness in the process, a single genotype can produce different assemblies (i.e., phenotypes) [9]. To estimate their frequencies, the self-assembly simulation was repeated 5000 times per genotype. When processing the outputs, two phenotypes were considered identical if they are rotations or translations of one another (but not mirror images) [15]. Further, a single ‘undefined’ placeholder phenotype was used for assemblies exceeding the maximum assembly size, as well as assemblies appearing <50 times in the assembly process, whose ensemble frequencies cannot be estimated reliably. While repeating the analysis with cut-offs of 25 and 100 gave consistent qualitative conclusions (see section E in S1 Text), the limited number of 5000 repetitions is a caveat, especially for the characterisation of low-frequency phenotypes.

**Fig 11:**
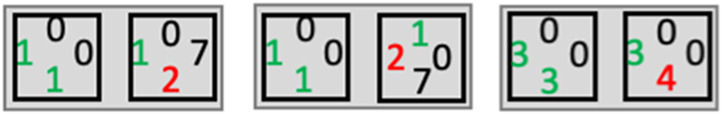
Assembly graph formalism (following [57]): These three sets of tiles all belong to the same assembly graph: after tile rotations, they all correspond to a case where the lower edge on the second tile binds three other edges (left edge on second tile, left or lower edge on first tile). To emphasise the matching binding properties, binding partners are colour-coded as red/green.

To generate the full ND GP map, one would need to run 5000 × *K*^*L*^ assemblies. To reduce computational costs, the assembly graph formalism was used: an assembly graph represents the binding properties of a set of tiles, such that two genotypes with the same assembly graph produce the same ensemble of phenotypes [57], see Fig 11. Thus, assembly was simulated only for one genotype per assembly graph, saving in computational costs in the assembly process. To identify genotypes with the same assembly graph, I went through each genotype in the map, generated its assembly graph and then compared it to previously encountered assembly graphs using NetworkX’s [58] graph isomorphism test. To speed up this process, genotypes differing by simple assembly-graph-preserving operations (swaps and rotations of tiles, re-labelling of non-binding edges to 0 and relabelling binding edges in ascending order, see [56]) were directly labelled as belonging to the same assembly graph.

While the assembly graph formalism makes a full analysis of the ND GP map computationally feasible, it comes with the caveat that sampling errors in a genotype’s ensemble frequencies are propagated to further genotypes with the same assembly graph.

### 4.4. Undefined phenotypes

Just as in previous deterministic GP maps [4], an undefined phenotype exists in several ND GP map models: an unfolded RNA chain in the RNA model and an undefined placeholder in the Polyomino model. Moreover, in the deterministic version of the synthetic GP map, which maps each genotype $\vec{g}$ to the lowest-$G_{p,\vec{g}}$ phenotype, the undefined phenotype was used when the two lowest-$G_{p,\vec{g}}$ phenotypes differ by less than 10^−4^, in line with conventions for ties in the lattice protein model [18]. These undefined structures were not included in the GP map characterisation since they are thought to be artefacts: for RNA, the high prevalence of the unfolded structure is due to the short sequence length of *L* = 12 [4], and in the Polyomino self-assembly, the unfolded structure is a placeholder for both unbounded and rare assemblies and should not be treated as a single phenotype. Thus, the sums $\sum_{p}$ in Table 1 were taken over all phenotypes except the undefined one, and quantities like phenotypic frequency and robustness are not reported for the undefined phenotype. This convention may be the reason why the RNA maps contain more low-robustness-low-evolvability genotypes in Fig 7 than found in a previous analysis [1].

## Supporting information

S1 TextTheory for robustness and evolvability in ND GP maps; Connection between $G_{p,\vec{g}}$ and $P(p|\vec{g})$ in the synthetic model; NC fragmentation in the ND GP maps; Applying theoretical bounds to the robustness-frequency data; Sensitivity to ensemble cut-off in the Polyomino model; Deterministic version of the synthetic GP map; Modified versions of the synthetic ND GP map model; Threshold-based framework for ND GP maps.(PDF)
