# Supplementary material for: A simple model captures key characteristics of biological non-deterministic genotype-phenotype maps
Source: PLoS Comput Biol. 2026 May 22;22(5):e1014272. doi: 10.1371/journal.pcbi.1014272 (PMC13196979; doi:10.1371/journal.pcbi.1014272)
Supplement: S1 Text — (PDF) [file pcbi.1014272.s001.pdf]

Supplementary Information: A simple model captures key characteristics of biological non-deterministic genotype-phenotype maps

Nora S. Martin

Contents

A Theory for robustness and evolvability in ND GP maps **1**
A.I Implications of genetic correlations in ND GP maps 1
A.II Limit on robustness and evolvability on the genotypic level 2

B Connection between $G_{p,\vec{g}}$ and $P(p|\vec{g})$ in the synthetic model **3**

C NC fragmentation in the ND GP maps **4**

D Applying theoretical bounds to the robustness-frequency data **5**

E Sensitivity to ensemble cut-off in the Polyomino model **6**

F Deterministic version of the synthetic GP map **7**

G Modified versions of the synthetic ND GP map model **9**

H Threshold-based framework for ND GP maps **11**

A Theory for robustness and evolvability in ND GP maps

A.I Implications of genetic correlations in ND GP maps

A GP map has positive genetic correlations if it has local correlations in genotype space: in the deterministic case, this means that for a genotype mapping to p, its mutational neighbours are more likely to map to p than an arbitrary genotype h [1]. Since the probability that mutational neighbours conserve p is given by robustness ρ_p, and the probability that an arbitrary genotype gives p is given by the frequency f_p, genetic correlations can be detected by analysing if the robustness ρ_p of a phenotype p exceeds its frequency f_p [1].

In ND GP maps, 'genetic correlations' have been defined by analogy: a GP map has genetic correlations if $\tilde{\rho}_p > \tilde{f}_p$ for all/most phenotypes p [2]. This can similarly be interpreted in terms of 'clustering' of p in genotype space: the following argument demonstrates that $\tilde{\rho}_p > \tilde{f}_p$ means that there is a positive covariance between $P(p|g)$ and $P(p|g')$, where g' is a mutational neighbour of g. To show this, let us begin by using the definition of $\tilde{\rho}_p$, and writing $\tilde{\rho}_p > \tilde{f}_p$, as:

$$\frac{1}{K^L \cdot \tilde{f}_p \cdot (K-1)L} \sum_g P(p|g) \sum_{g' \in \mathcal{N}_g} P(p|g') > \tilde{f}_p$$

Rearranging gives:

$$\frac{1}{K^L \cdot (K-1)L} \sum_{g, g' \in \mathcal{N}_g} P(p|g) P(p|g') > \tilde{f}_p^2$$

The expression on the LHS can be recognised as the mean of $P(p|g)P(p|g')$, averaged over all pairs of a genotype g and its mutational neighbour g'. Similarly, on the RHS, $\tilde{f}_p$ is the average of $P(p|g)$ over all genotypes g (or, equivalently over all mutational neighbours of all genotypes). This gives:

$$E[P(p|g)P(p|g')] > E[P(p|g)]E[P(p|g')]$$

And thus, a positive covariance between $P(p|g)$ and $P(p|g')$:

$$cov(P(p|g), P(p|g')) = E[P(p|g)P(p|g')] - E[P(p|g)]E[P(p|g')] > 0$$

Therefore, genetic correlations can be interpreted as a type of 'local clustering' in genotype space: if a genotype g has a high/low $P(p|g)$ (relative to the mean prevalence of p, which is given by its frequency $\tilde{f}_p$), then its mutational neighbours g' are more likely to also have high/low $P(p|g')$ than in a completely uncorrelated GP map.

This perspective on genetic correlations in ND GP maps clarifies the previously noted [2] connection between 'genetic correlations' and Ancel & Fontana's [3] concept of plastogenetic congruence. Plastogenetic congruence can be defined in several ways, for example as follows: let us consider a genotype g, for which phenotype p is the highest-$P(p|g)$ phenotype in the ensemble. Then, in a GP map with plastogenetic congruence, g's mutational neighbours g' are likely to retain p as a high-$P(p|g)$ phenotype, even if not as the *highest*-$P(p|g)$ phenotype. While this definition differs from that of genetic correlations, for example with its focus on the *highest*-$P(p|g)$ phenotype for a given genotype, it is clearly related.

A.II Limit on robustness and evolvability on the genotypic level

To put limits on genotypic robustness and evolvability in ND GP maps, let us exploit parallels with simpler GP maps without ND. In GP maps without ND, there is a simple trade-off between robustness and evolvability on the genotypic level [4]: each genotype has $(K-1)L$ neighbours, where K is the alphabet size and L the sequence length. Each neighbour can either be neutral and contribute $\Delta\rho_g = 1/((K-1)L)$ to the genotypic robustness, or be non-neutral. If the neighbour is non-neutral and produces a phenotype that is not already in the neighbourhood, the mutation can contribute $\Delta\epsilon_g = 1$ to the evolvability, otherwise the mutation contributes to neither robustness nor evolvability. Thus, the following bound applies to GP maps without ND [4] (in the following, quantities without a tilde belong to deterministic GP maps):

$$\epsilon_g + ((K-1)L)\rho_g \leq (K-1)L$$
$$\frac{\epsilon_g}{(K-1)L} + \rho_g \leq 1 \qquad \text{(A)}$$

To apply this result to GP maps *with* ND, let us use parallels between genotypic robustness and evolvability in GP maps *with* and *without* ND: in a GP map with ND, each genotype maps to a distribution of phenotypes. Let us now construct a GP map without ND, by simply drawing a single phenotype from each genotype's ensemble and recording it. If a large number of such 'frozen' GP maps without ND is drawn from a GP map with ND, then the average robustness of a genotype over the set of 'frozen maps' $\langle\rho_g\rangle_{\text{frozen}}$ approximates its robustness in the full ND GP map $\tilde{\rho}_g$ [2]. The same approximate equivalence holds for genotypic evolvability [2]. This equivalence holds by design, thereby ensuring consistency between GP maps with and without ND.

The approximate equivalence between ND GP maps and averages over specific GP maps without ND can now be applied to eq. A: in each of the frozen maps without ND, the genotypic robustness ρ_g and evolvability ϵ_g must satisfy $\frac{\epsilon_g}{(K-1)L} + \rho_g \leq 1$. Thus, the same inequality must hold for their mean over frozen maps:

$$\langle \frac{\epsilon_g}{(K-1)L} + \rho_g \rangle_{\text{frozen}} \leq 1$$
$$\frac{\langle\epsilon_g\rangle_{\text{frozen}}}{(K-1)L} + \langle\rho_g\rangle_{\text{frozen}} \leq 1$$

Then the approximate equivalence of the means over frozen maps ($\langle\epsilon_g\rangle_{\text{frozen}}$ and $\langle\rho_g\rangle_{\text{frozen}}$) with the ND GP map quantities ($\tilde{\epsilon}_g$ and $\tilde{\rho}_g$) can be applied to write:

$$\frac{\tilde{\epsilon}_g}{(K-1)L} + \tilde{\rho}_g \leq 1$$

Thus, genotypic robustness and evolvability in the ND GP map approximately satisfy the same trade-off as in the well-known case without ND.

B Connection between $G_{p,\vec{g}}$ and $P(p|\vec{g})$ in the synthetic model

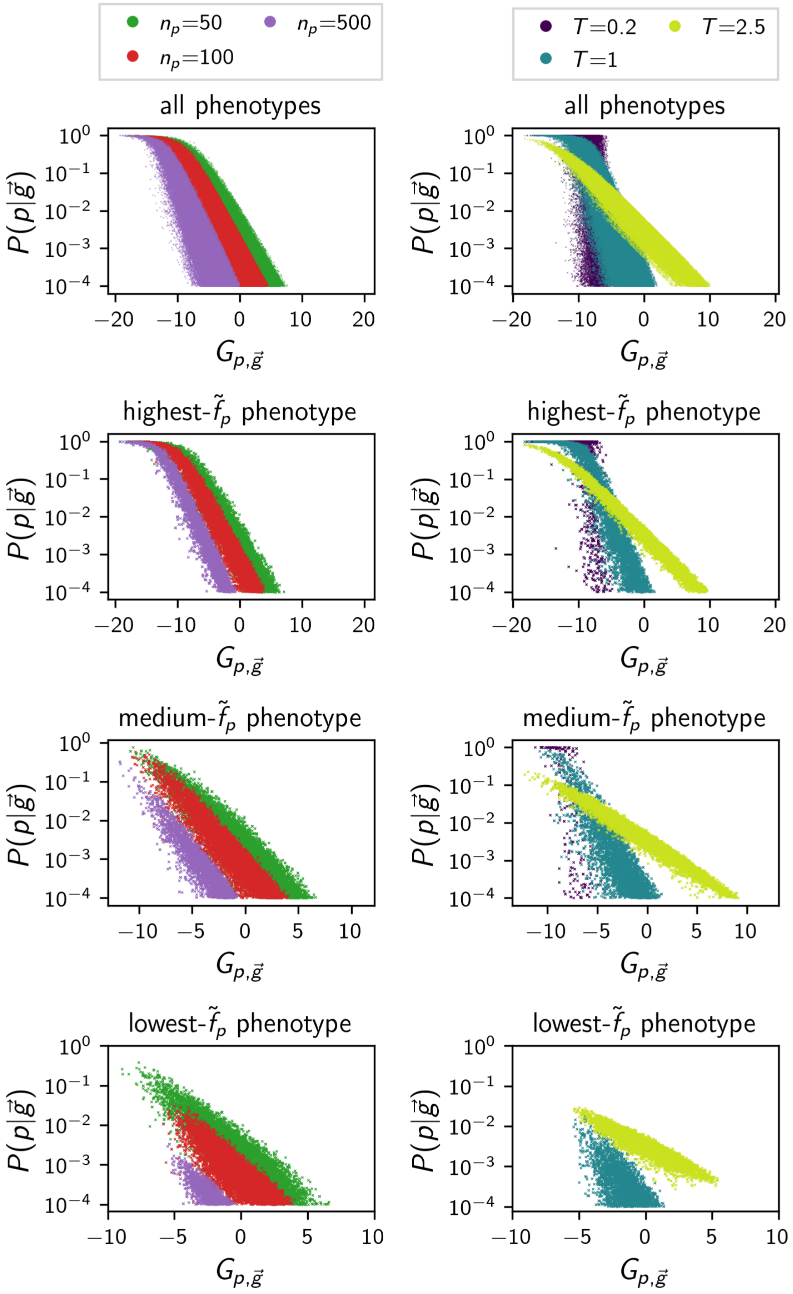

Figure A: ***Relationship between $G_{p,\vec{g}}$ and $P(p|\vec{g})$ in the synthetic model:*** *(first row) $P(p|\vec{g})$ is plotted against $G_{p,\vec{g}}$ for all phenotypes p in 10^4 randomly generated genotypes g. (second row) The data for only one phenotype - the one with highest $\tilde{f}_p$ - is shown, to account for the phenotype-dependence of $G_{p,\vec{g}}$ and $P(p|\vec{g})$. (third row) Same for a medium-$\tilde{f}_p$ phenotype. (fourth row) Same for the lowest-$\tilde{f}_p$ phenotype. Low-$G_{p,\vec{g}}$ values imply high-$P(p|\vec{g})$ in all cases, as expected from eq. 2. The same models as in main text Fig 4 are shown (see legends at the top of each column).*

In the main text, genetic correlations in the synthetic model were explained as follows: If phenotype p has low $G_{p,\vec{g}}$ for genotype $\vec{g}$, p is also likely to have low $G_{p,\vec{g}'}$ for genotypes $\vec{g}'$ that are one point mutation away from $\vec{g}$. Since $G_{p,\vec{g}}$ influences the ensemble probability via eq. 2, this implies correlations in ensemble probabilities and thus genetic correlations. Fig A investigates this second step more closely: the connection between $G_{p,\vec{g}}$

and $P(p|\vec{g})$. According to eq. 2, $P(p|\vec{g})$ depends on $G_{p,\vec{g}}$, but also on the $G_{q,\vec{g}}$ of other phenotypes q through the normalisation. This is consistent with Fig A, which illustrates that there is no one-to-one dependence between $G_{p,\vec{g}}$ and $P(p|\vec{g})$, but that low-$G_{p,\vec{g}}$ values tend to imply high $P(p|\vec{g})$ and vice versa.

C NC fragmentation in the ND GP maps

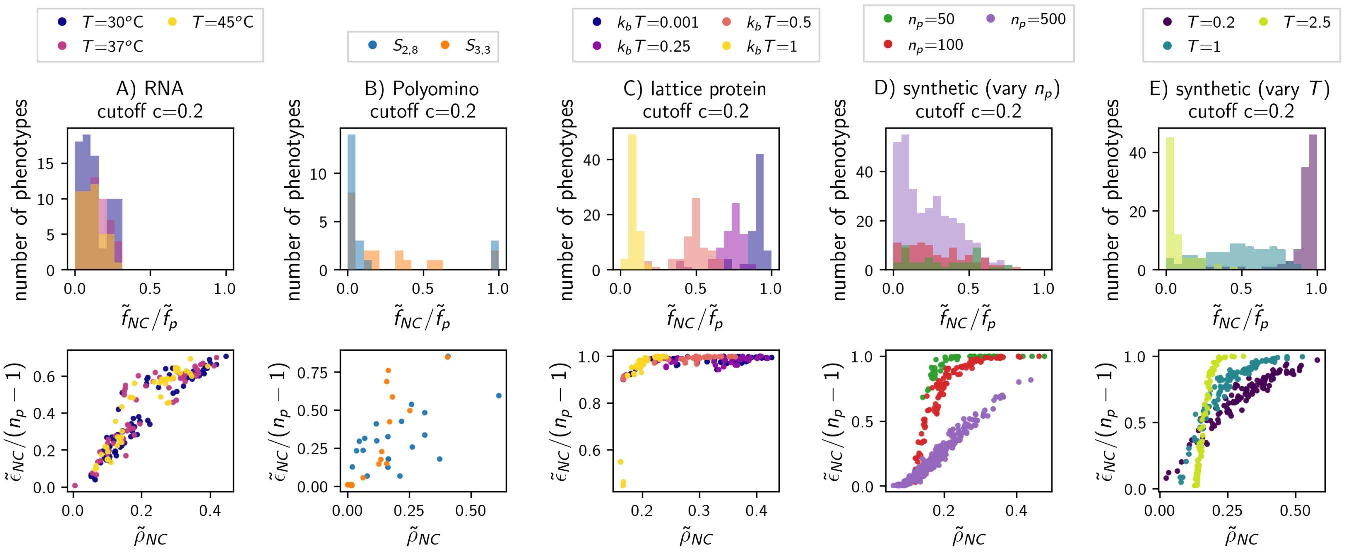

Figure B: ***Robustness and evolvability at the level of neutral components:*** *Here, two genotypes with p in their ensembles are considered to belong to the same neutral component only if these genotypes are connected by a mutational path along which $P(p|g)$ never drops below 0.2. (first row) For each phenotype, the ratio between the frequency of the highest-frequency neutral component and the full phenotypic frequency is computed and shown as a histogram. In many cases, these fractions are smaller than one, showing that the neutral component level is distinct from the phenotypic level. (second row) The robustness-evolvability relationship is plotted for the highest-frequency neutral component of each phenotype. Phenotypes are included only if they have at least genotype with $P(p|g) > 0.2$ and thus one or more NCs above the threshold.*

In the main text, robustness and evolvability were defined on both the genotypic and the phenotypic level. However, in the case of deterministic GP maps, there is a third evolutionarily relevant scale on which these features can be defined: the level of neutral components (NCs), i.e. networks of genotypes that share a single phenotype p and that are mutationally connected [5]. During evolution under stabilising selection on a neutral component, a population can only encounter phenotypes in the mutational neighbourhood of that neutral component [5]. Thus, the neutral component evolvability, the number of different phenotypes mutationally accessible from that neutral component, can be relevant.

In ND GP maps, where every genotype maps to a set of categorical phenotypes and an ensemble probability for each phenotype, the concept of neutral components becomes less clear-cut. Defining neutral components in such maps requires us to coarse-grain incremental differences in the ensemble probabilities to categorical information, to obtain sets of "phenotypically equivalent" genotypes, which populations can access under stabilising selection for a certain phenotype p. Then, neutral components would correspond to connected network components out of such sets of "phenotypically equivalent" genotypes.

There are several possible ways of defining "phenotypically equivalent" genotypes in ND GP maps, and the appropriate choice will depend on the scenario being modelled. Here, I simply chose a scenario of threshold-based selection, for example where the correctly folded state of a molecule needs to exceed a certain concentration. Thus, the criterion for "phenotypically equivalent" genotypes of phenotype p becomes $P(p|g) \geq c$ for some concentration c (see also [6]). This then defines a set of neutral components for each phenotypes: mutationally connected networks of genotypes which all have $P(p|g) \geq c$. On this neutral component level, frequency, robustness and evolvability can be computed in analogy to their phenotypic counterparts, but with the sum/product taken only over the respective neutral component NC instead of all

genotypes:

$$\tilde{f}_{\mathrm{NC}} = \frac{1}{K^L} \sum_{g \in \mathrm{NC}} P(p|g) \tag{B}$$

$$\tilde{\rho}_{\mathrm{NC}} = \frac{1}{K^L \cdot \tilde{f}_{\mathrm{NC}} \cdot (K-1)L} \sum_{g \in \mathrm{NC}} P(p|g) \sum_{g' \in \mathcal{N}_g} P(p|g') \tag{C}$$

$$\tilde{\epsilon}_{\mathrm{NC}} = \sum_{p' \neq p} \left(1 - \Pi_{g \in \mathrm{NC}} \Pi_{g' \in \mathcal{N}_g} (1 - P(p'|g')P(p|g))\right) \tag{D}$$

Fig B illustrates what the robustness-evolvability relationship would look like on the neutral component level, focussing on a single neutral component for each phenotype, the one with the largest frequency $\tilde{f}_{\mathrm{NC}}$. A relatively high cut-off of $c = 0.2$ was chosen, in order to gain a clear contrast with the phenotypic level, which corresponds to $c = 0$. The contrast is reflected in the neutral component frequencies $\tilde{f}_{\mathrm{NC}}$, which only cover a small fraction of the full phenotypic frequencies $\tilde{f}_p$ in many maps (top row of Fig B). Turning to robustness and evolvabilities of these neutral components, Fig B shows that the robustness-evolvability relationship remains non-negative on the neutral component level, as before on the phenotypic level. This is consistent with results for *deterministic* GP maps, where NC evolvability and robustness are positively correlated [5].

D Applying theoretical bounds to the robustness-frequency data

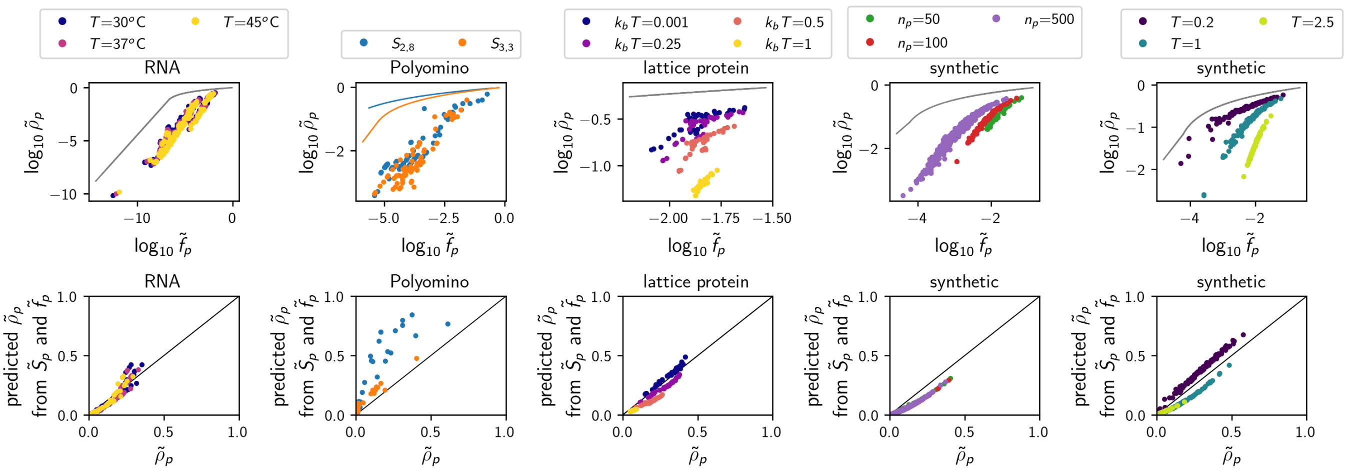

Figure C: ***Applying Sappington & Mohanty's [7] theory on phenotypic robustness: (first row)*** *Logarithmic plot of phenotypic robustness $\tilde{\rho}_p$ against phenotypic frequency $\tilde{f}_p$ (scatter points, shown for all phenotypes with $\tilde{\rho}_p > 0$ and $\tilde{f}_p > 0$), together with Sappington & Mohanty's [7] upper bound (drawn as a line). Note that the two versions of the Polyomino model have different upper bounds since they differ in their alphabet size K and sequence length L.* ***(second row)*** *Sappington & Mohanty's [7] approximate relationship between phenotypic robustness $\tilde{\rho}_p$, phenotypic frequency $\tilde{f}_p$ and phenotypic entropy $\tilde{S}_p$ is tested: the predicted phenotypic robustness (RHS of eq. E) is plotted against the computational phenotypic robustness $\tilde{\rho}_p$. The black line illustrates $x = y$, i.e. a perfect prediction. The RNA model was already analysed by Sappington & Mohanty [7], and is included for completeness.*

A recent theoretical paper proposed approximations for the phenotypic-robustness-frequency data in ND GP maps [7], thus providing another topic where the ND GP maps from the present paper can be applied: The first approximation provides an upper bound for the phenotypic robustness $\tilde{\rho}_p$ of phenotypes of a given frequency $\tilde{f}_p$. This upper bound is plotted together with the simulation data in the upper row of Fig C. The proposed upper bound is consistent with the data, despite the approximations made in its derivation (esp. a binary assumption about phenotypic ensemble frequencies: that each phenotype either has a fixed ensemble frequency x or does not exist in an ensemble). The second approximation concerns the relationship between

phenotypic robustness $\tilde{\rho}_p$, phenotypic frequency $\tilde{f}_p$ and the entropy of a phenotype $\tilde{S}_p$ [7]:

$$\tilde{\rho}_p \approx \frac{K^L \tilde{f}_p \tilde{S}_p \exp(-\tilde{S}_p)}{L \log(K)} \tag{E}$$

Here, the entropy $\tilde{S}_p$ quantifies whether the probability weight of a phenotype p is concentrated on a small or large number of genotypes and is defined as [7]:

$$\tilde{S}_p = - \sum_{\text{genotypes } g} \frac{P(p|g)}{\tilde{f}_p K^L} \log \frac{P(p|g)}{\tilde{f}_p K^L} \tag{F}$$

To test the relationship from eq. E, the predicted phenotypic robustness (RHS of eq. E) is plotted against the actual phenotypic robustness in the maps, $\tilde{\rho}_p$ (second row of Fig C), finding a good correlation in agreement with the original test data esp. on RNA [7]. However, for the synthetic ND GP map, the prediction tends to overestimate robustness in low-T maps and underestimate robustness in high-T maps, which may be because the assumptions underlying the calculations are an approximation to full ND GP maps (for example, that $P(p|g)$ only takes one non-zero value for each p).

E Sensitivity to ensemble cut-off in the Polyomino model

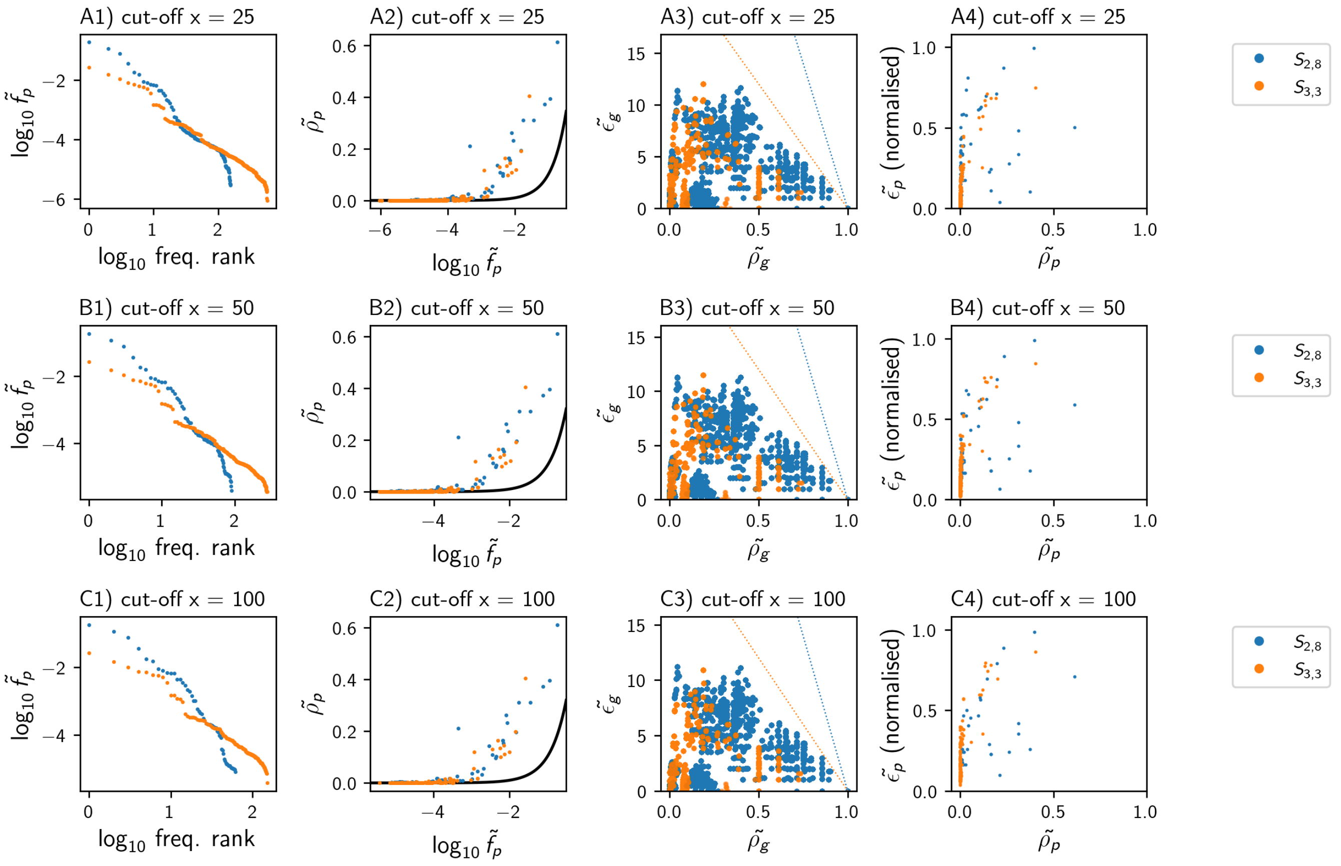

Figure D: ***Polyomino ND GP map analysis for different thresholds x in the ensemble calculation:*** *The ensemble frequencies in the Polyomino model are based on the assembled structures found after 5000 simulated assembly processes per assembly graph. Since the frequencies of low-$P(p|g)$ structures are difficult to estimate reliably, assemblies appearing fewer than x times were treated as 'undefined'. To analyse the impact of this choice, this figure repeats the ND GP map analysis for different choices of x, one in each row:* ***(A)*** *x = 25,* ***(B)*** *x = 50 (as in the main text, shown for context),* ***(C)*** *x = 100.*

In the Polyomino model, ensemble frequencies were estimated from a finite number of self-assembly simulations: the self-assembly process was repeated 5000 times and thus the ensemble frequency was estimated to

be $P(p|g) = x/5000$ for a structure p appearing x times. However, since reliable estimates cannot be made for structures appearing very rarely, a cut-off is applied and structures with less than $x = 50$ appearances are treated as 'undefined' phenotypes (see methods section in the main text). To investigate the impact this cut-off may have on the ND GP map analysis, the ND GP map analysis from the main text is repeated for two different cut-offs, $x = 25$ and $x = 100$ (shown in Fig D, with the $x = 50$ data also shown for comparison). On a qualitative level, the conclusions are unaffected.

F Deterministic version of the synthetic GP map

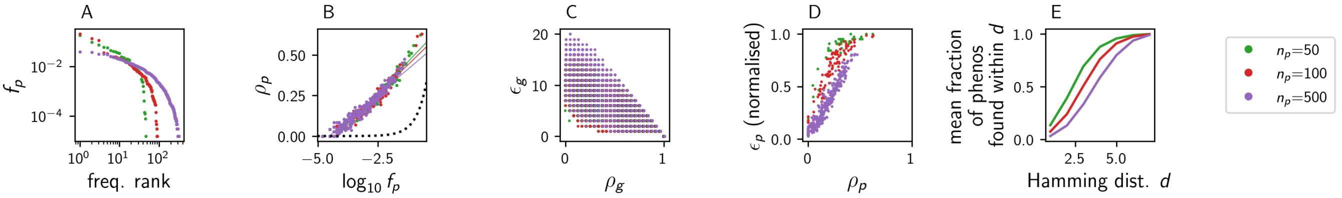

Figure E: ***The synthetic GP map in its deterministic limit*** *($T = 0$)* ***displays the shared properties of GP maps without ND (see review [8]): (A)*** *The relationship between phenotypic frequency f_p and frequency rank shows clear phenotypic bias.* ***(B)*** *Phenotypic robustness ρ_p is plotted against frequency f_p on a lin-log scale, showing genetic correlations ($\rho_p > f_p$ for the majority of phenotypes, highlighted by the black dotted line showing $\rho_p = f_p$), and a good fit with a log-linear function (fitted lines).* ***(C)*** *There is a trade-off between genotypic evolvability ϵ_g and robustness ρ_g (x-values are shifted by up to $0.1/((K-1)L)$ to show overlapping data points more clearly).* ***(D)*** *There is a positive trend in the relationship between phenotypic robustness ρ_p and evolvability ϵ_p (here normalised by the maximum possible evolvability $m_p - 1$, where $m_p \leq n_p$ is the number of phenotypes that appear at least once in the deterministic GP map).* ***(E)*** *Shape-space covering (following convention of ref [9]): how many distinct phenotypes are present within a Hamming distance d from a given initial genotype? Here, the mean over 10^2 randomly selected initial genotypes is shown, normalised by the highest possible value of $m_p - 1$. For the phenotypic quantities (f_p, ρ_p and ϵ_p), phenotypes are included if they appear at least once in the GP map.*

The synthetic model, in addition to being a simple ND GP map, also defines a GP map without ND in

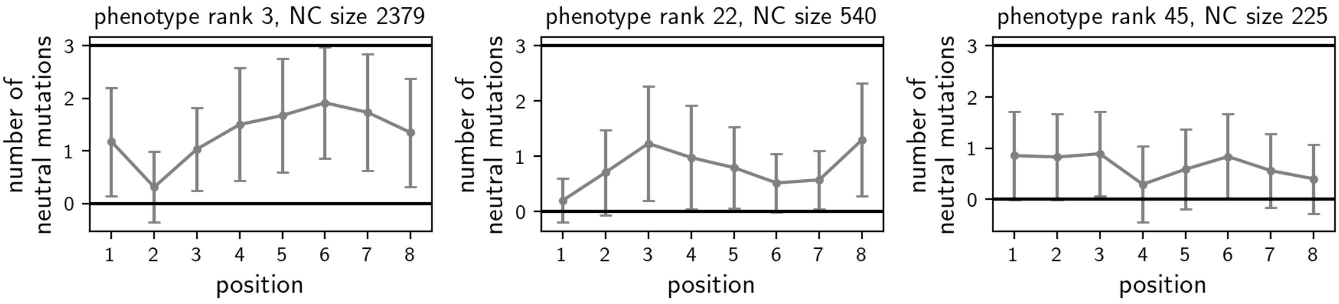

Figure F: ***Does the synthetic GP map in its deterministic limit*** *($T = 0$,* ***here for*** *$n_p = 100$)* ***have position-specific sequence constraints:*** *sequence constraints are visualised for three phenotypes of different frequency ranks, one phenotype in each subplot (see titles). To quantify sequence constraints computationally, I first selected a random genotype g mapping to p and extracted the corresponding neutral component (NC), the set of genotypes mapping to p that are accessible through neutral mutations from g. Then, following Weiß & Ahnert [10], I counted the possible neutral mutations at each sequence position position as one indicator of sequence constraints. For each position (x-axis), the mean and standard deviation over all genotypes in the NC are shown (y-axis). The plots show that different sequence positions in a NC have different mean sequence constraints, but there is considerable variability around this mean, even within a single NC.*

the deterministic limit $T \to 0$. In this deterministic GP map, each genotype $\vec{g}$ maps to the phenotype p with lowest $G_{p,\vec{g}}$; in the exceptional case that the two lowest-$G_{p,\vec{g}}$ phenotypes differ by less than 10^{-4}, the

genotype is treated as 'undefined', in line with conventions for ties in the HP model [1]. Thus, for a given sequence length L, only one free parameter remains: the number of phenotypes n_p. However, one change was made to the simple model introduced in the main text: the alphabet size was raised to $K = 4$ (with sequence length $L = 8$), to obtain a higher number of mutations per sequence position and thus a more nuanced picture of sequence constraints. In order to generalise the model to an arbitrary alphabet size K, the parameter vectors $\vec{v_p}$ are extended to length KL and the genotypes constructed from an alphabet (0, 1, ... K-1). Then, $G_{p,g}$ can be computed as:

$$G_{p,g} = \sum_{\text{seq. positions } i} (\vec{v_p})_{i+\vec{g}_i L}$$

Fig E analyses three versions of this GP map, for $n_p = 50$, $n_p = 100$ and $n_p = 500$, finding all central shared features of deterministic GP maps (as reviewed in [8]): first, there is strong phenotypic bias, since different phenotypes differ in their phenotypic frequencies f_p by orders of magnitude (Fig EA). Secondly, the phenotypic frequency-robustness relationship is well-described by a log-linear fit (Fig EB) and the phenotypic robustness is higher than the frequency for the majority of phenotypes (exceptions: zero-robustness phenotypes generated by a very low number of genotypes with $f_p \leq 4/K^L$; many of these phenotypes are only generated by a single genotype and thus cannot show genetic correlations). Thirdly, the relationship between *genotypic* evolvability and robustness shows a trade-off (Fig EC), but that of *phenotypic* evolvability and robustness is positive (Fig ED). Finally, the synthetic model satisfies the space-shape-covering property, i.e. most phenotypes are available within a small Hamming distance from an arbitrary genotype (Fig EE). Thus, in the deterministic limit, the synthetic GP map displays the well-studied shared characteristics of deterministic GP maps.

Simple models reproducing these shared characteristics and approximating GP maps have typically had variable sequence constraints as a key feature: each sequence position is characterised by a fixed capacity for phenotype-conserving mutations, which only depends on the phenotype or at most on the neutral component (NC) [10–15]. This prompts the question of whether the synthetic GP map similarly has variable sequence constraints as a defining feature. Fig F visualises these sequence constraints for three phenotypes. The sequence constraints, quantified by the mean number of neutral mutations, show some position-dependence, but there is considerable variation around the mean. Thus, a model that mainly uses mean sequence constraints to characterise a NC may fail to approximate this GP map and its structure well. However, the question of when a 'sequence-constraint-based' model stops being a useful approximation needs further investigation: Some variation around the mean can be accounted for within the framework of 'sequence-constraint-based' models, for example when extrapolating from sequence constraints to NC sizes [12]. Small differences in sequence constraints are also present in an existing toy model called the 'RNA-like GP map model' [16].

The error bars in Fig F may be interesting themselves since they represent a type of epistasis: the number of neutral mutations at a particular site is highly genotype-dependent, even among genotypes in a single NC. This epistasis may be of biological relevance: some variability in the position-specific sequence constraints is already present in the RNA GP map [12], even though this GP map has been approximated based on its sequence constraints [12, 14]. Moreover, empirical evidence [17] and computational models fitted to sequence families [18] suggest that mutations, which are only neutral for some genotypes in a sequence family, may not be rare.

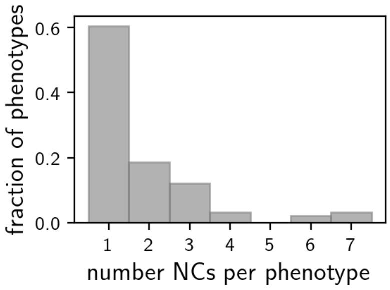

Figure G: ***NC fragmentation in the synthetic GP map in its deterministic limit ($T = 0$, here for $n_p = 100$):*** *A neutral space is fragmented when there is more than one disjoint NC per phenotype.*

Going beyond the scale of a NC, there is an additional feature that the synthetic GP map shares with

biophysical models like RNA: the fact that the set of genotypes mapping to a single phenotype can consist of several mutationally disconnected subsets or NCs (Fig G). This arises from another type of epistasis between neutral mutations [5], where single mutations are non-neutral, but pairs of mutations can rescue the phenotype, leading to two or more disjoint subnetworks for a given phenotype. This epistasis is typically absent in the simple sequence-constraint-based toy models.

Thus, the synthetic model produces a deterministic GP map with neutral epistasis. This observation is in line with the fact that other models, which similarly combine additive functions with non-linearities, are known produce epistasis in a different kind of landscape: continuos landscapes derived directly from ensemble probabilities [19].

G Modified versions of the synthetic ND GP map model

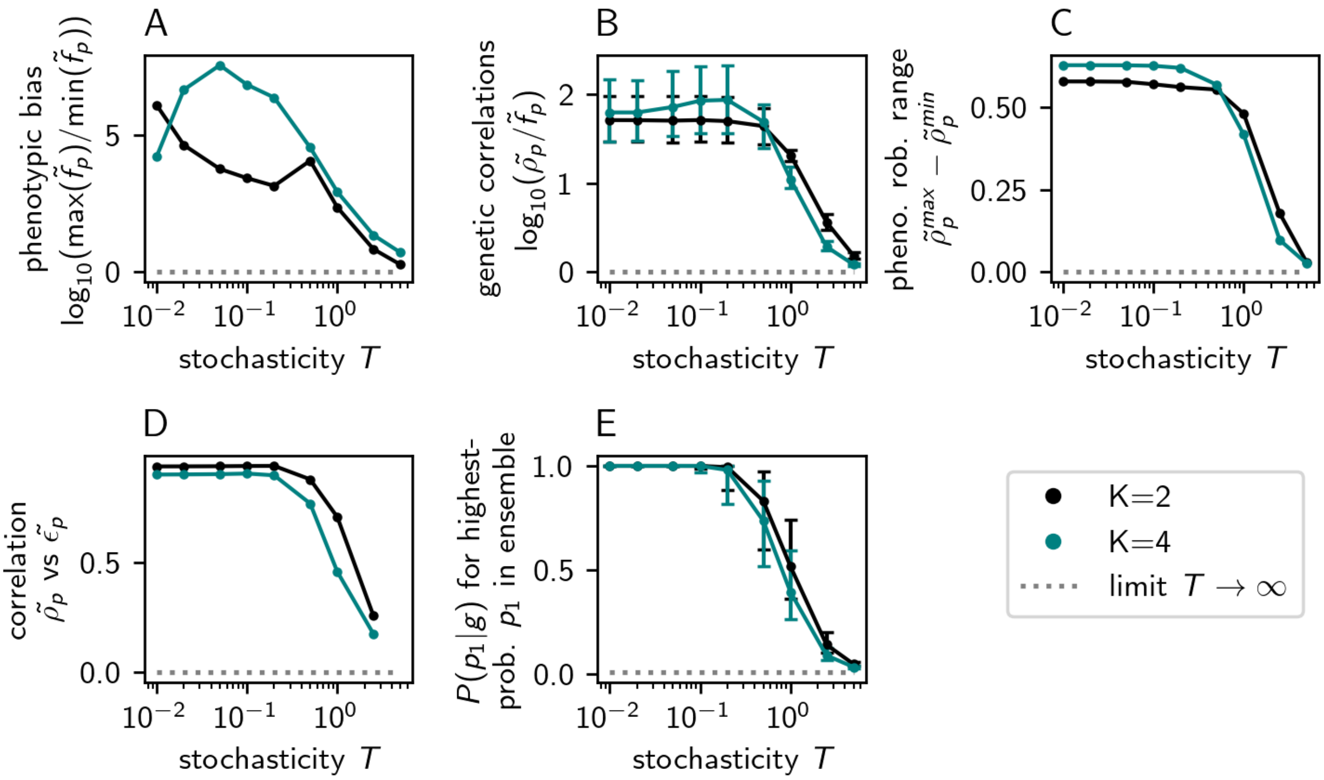

Figure H: ***ND GP map analysis of an alternative version of the synthetic ND GP map built with an alphabet size of*** $K = 4$***:*** *The analysis follows Fig 8 in the main text (all with* $n_p = 100$*):* ***(A)*** *Log-ratio of the highest to lowest phenotypic frequencies as a proxy for phenotypic bias.* ***(B)*** *Log-ratio of phenotypic robustness to frequency as a proxy for genetic correlations (median and quartiles over all phenotypes with* $\tilde{f}_p > 0$ *and* $\tilde{\rho}_p > 0$ *shown).* ***(C)*** *Difference between minimum and maximum phenotypic robustness* $\tilde{\rho}_p$ *in the map.* ***(D)*** *Pearson correlation coefficient between robustness and evolvability on the phenotypic level (no value is computed if maximum and minimum evolvability values differ by less than* 10^{-6}*).* ***(E)*** *Typical* $P(p|\vec{g})$ *of highest-ranking phenotype p in the ensemble of a given genotype* $\vec{g}$ *(median and quartiles over all genotypes shown).*

In the main text, I analysed synthetic models constructed with alternative definitions: I found that some models built with other non-linear functional forms, as well as alternative initialisation schemes of the parameter vectors $\vec{v}_p$ also generate the following ND GP map properties: strong phenotypic bias, genetic correlations and a positive phenotypic robustness-evolvability relationship. This section analyses two further alternative synthetic models: one with an alphabet size of $K = 4$ in Fig H, and one case, where *both* the functional form and the parameter initialisation are changed in Fig I.

The alphabet size in the synthetic model can easily be adjusted, see section F. Thus, I compared a map built with the DNA/RNA alphabet size of $K = 4$ to the binary alphabet of $K = 2$ used in the main text.

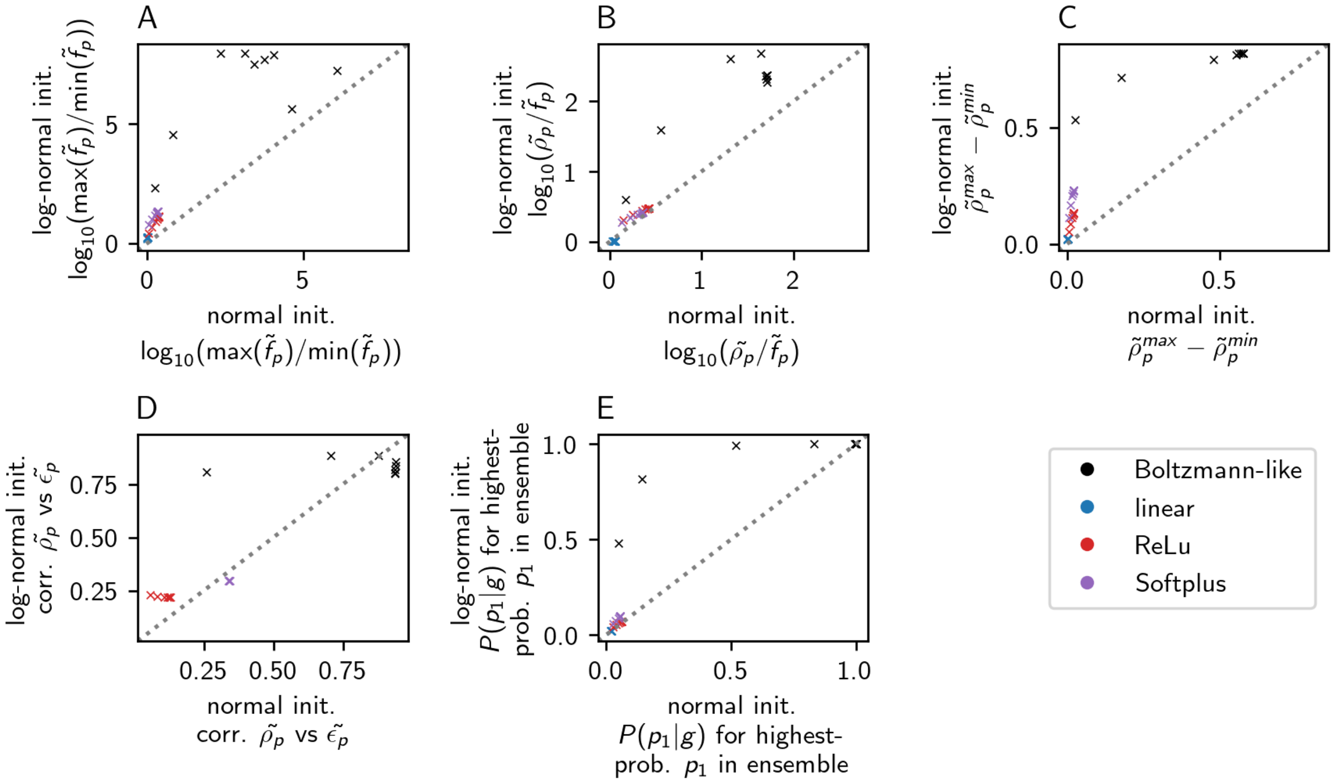

Figure I: ***ND GP map analysis of synthetic ND GP maps combining alternative functional forms with a log-normal initialisation:*** *This figure compares the features of ND GP maps initialised with a log-normal distribution (y-axis) to those initialised with a normal distribution (x-axis). This comparison is made for models built with the Boltzmann-like, 'linear', ReLu and Softplus functional forms defined in the main text, each for nine values of T (0.01, 0.02, 0.05, 0.1, 0.2, 0.5, 1, 2.5, 5), all with $n_p = 100$. The following features are compared:* ***(A)*** *Log-ratio of the highest to lowest phenotypic frequencies as a proxy for phenotypic bias.* ***(B)*** *Log-ratio of phenotypic robustness to frequency as a proxy for genetic correlations (median over all phenotypes with $\tilde{f}_p > 0$ and $\tilde{\rho}_p > 0$).* ***(C)*** *Difference between minimum and maximum phenotypic robustness $\tilde{\rho}_p$ in the map.* ***(D)*** *Pearson correlation coefficient between robustness and evolvability on the phenotypic level (no value is computed if maximum and minimum evolvability values differ by less than 10^{-6}).* ***(E)*** *Typical $P(p|\vec{g})$ of highest-ranking phenotype p in the ensemble of a given genotype $\vec{g}$ (median over all genotypes).*

By adjusting the sequence length in the $K = 4$ map to $L = 8$, I kept the total number of genotypes K^L close to the 2^{15} genotypes in the binary case. The analysis of both maps in Fig H suggests that changing the alphabet size does not have an impact on central ND GP map features: both maps display strong phenotypic bias, genetic correlations and a positive phenotypic robustness-evolvability relationship.

Having adjusted several aspects of the synthetic model in isolation, I finally investigated a model with two modifications compared to the initial definition: a functional form approximating a linear function combined with a lognormal distribution for the parameter initialisation. This combination may be interesting since the functional forms from the 'linear' family (i.e. the shifted linear, ReLu and Softmax) produced ND GP maps close to the trivial $T \to \infty$ limit - with little phenotypic bias, low genetic correlation and little variation in phenotypic robustness. In contrast, switching the parameter initialisation to a log-normal distribution reinforced phenotypic bias. This raises the question of whether a log-normal parameter initialisation can 'rescue' the phenotypic bias in models in combination with functional forms from the 'linear' family. Fig I demonstrates that this hypothesised compensatory effect is only partially present and that typical ensembles continue to lack high-$P(p|g)$ phenotypes (Fig IE) and thus continue to resemble the high-T limit. However, it is possible that there exist other, carefully tuned initialisations that could rescue the GP map bias, both in individual ensembles and at the level of phenotypic frequencies.

H Threshold-based framework for ND GP maps

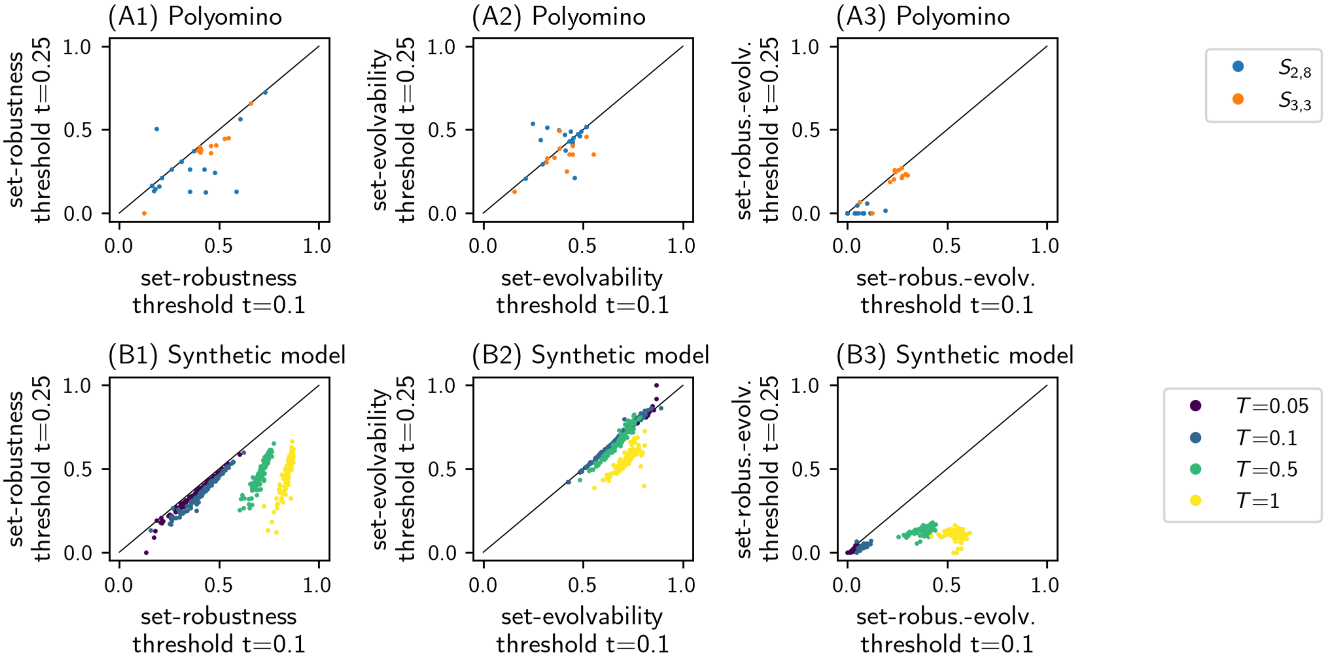

Figure J: ***Analysis of ND GP maps with a threshold-based framework [6]:*** *the threshold-based framework is applied to the Polyomino map, where it was first proposed* ***(row A)****, and to the synthetic map with* $n_p = 100$ ***(row B)****. Since this framework has a free parameter, the threshold t for including a phenotype in the 'set' of a genotype (if* $P(p|g) > t$*), the sensitivity to this threshold is tested: in* ***column 1****, the phenotypic set-robustness computed with* $t = 0.25$ *is plotted against that computed with* $t = 0.1$*, and similarly for the set-evolvability in* ***column 2*** *and the set-robustness-evolvability in* ***column 3****. Data is reported for phenotypes, which appear in at least one set, i.e. those with* $P(p|g) > t$ *for at least one genotype g.*

For completeness, let us consider an alternative way of characterising robustness and evolvability in ND GP maps: 'set-robustness' and 'set-evolvability' first proposed by Jouffrey, Leonard & Ahnert [6]. These quantities assume that there is a fixed probability cut-off above which phenotypes are relevant: all phenotypes above the cut-off are treated as equal (even if they have different ensemble frequencies), and all phenotypes below the cut-off are ignored. This was proposed in the context of gene duplication, and it was assumed that correctly folded structures will contribute to fitness if they are more frequent than a given cut-off, but that above this cut-off the exact value of their ensemble frequency is irrelevant. Since it is possible that a mutation both preserves one or more 'relevant' phenotypes, and introduces a new one, this cut-off-based metric can give a positive relationship between evolvability and robustness, even on a genotypic level [6].

Here, the concepts of phenotypic 'set-robustness', 'set-evolvability' and 'set-robust-evolvability' are applied to both the Polyomino ND GP map (where it was first proposed) and the synthetic ND GP map (Fig J). To apply these definitions to a model beyond the Polyomino model, these definitions need to be modified to be applied in ND GP maps that cannot be described as assembly graphs: rather than averaging over all genotypes with the same assembly graph, here the analysis averages over all genotypes for which the given phenotype p exceeds the cutoff.

Even for a single ND GP map with a fixed set of parameters, 'set-robustness', 'set-evolvability' and 'set-robust-evolvability' can be dependent on the choice of cut-off in their definitions, especially in the high-stochasticity limit. Stochasticity is likely to play a role due to its effect on the frequencies found in a typical ensemble: if typical ensemble frequencies are well above/below the cut-off (for example, in low-T ensembles, the most frequent phenotype has an ensemble frequency close to one, and the second-highest a much lower ensemble frequency), then the exact value of the cut-off may be less important.

Because of the potential cut-off dependence and the lack of a biologically motivated cut-off in our models, these set-based quantities are not used further to characterise ND GP maps. While this paper also uses a cut-off in the Polyomino model for computational reasons (see section E), this cut-off merely discards

phenotypes with low ensemble frequencies, but keeps the quantitative ensemble frequency information for all other phenotypes.

References

[1]S. F. Greenbury, S. Schaper, S. E. Ahnert, and A. A. Louis, "Genetic correlations greatly increase mutational robustness and can both reduce and enhance evolvability", PLOS Comput. Biol. **12**, e1004773 (2016).

[2]P. García-Galindo, S. E. Ahnert, and N. S. Martin, "The non-deterministic genotype–phenotype map of RNA secondary structure", J. R. Soc. Interface **20**, 20230132 (2023).

[3]L. W. Ancel and W. Fontana, "Plasticity, evolvability, and modularity in RNA", Journal of Experimental Zoology **288**, 242–283 (2000).

[4]A. Wagner, "Robustness and evolvability: a paradox resolved", Proc. R. Soc. Lond. B **275**, 91–100 (2008).

[5]S. Schaper, I. G. Johnston, and A. A. Louis, "Epistasis can lead to fragmented neutral spaces and contingency in evolution", Proc. R. Soc. Lond. B **279**, 1777–1783 (2012).

[6]V. Jouffrey, A. Leonard, and S. Ahnert, "Gene duplication and subsequent diversification strongly affect phenotypic evolvability and robustness", R. Soc. Open Sci. **8**, 201636 (2021).

[7]A. Sappington and V. Mohanty, "Probabilistic genotype-phenotype maps reveal mutational robustness of RNA folding, spin glasses, and quantum circuits", Phys. Rev. Research **7**, 013118 (2025).

[8]S. E. Ahnert, "Structural properties of genotype–phenotype maps", J. R. Soc. Interface **14**, 20170275 (2017).

[9]E. Ferrada and A. Wagner, "A comparison of genotype-phenotype maps for RNA and proteins", Biophys. J. **102**, 1916–1925 (2012).

[10]M. Weiss and S. E. Ahnert, "Neutral components show a hierarchical community structure in the genotype–phenotype map of RNA secondary structure", J. R. Soc. Interface **17**, 20200608 (2020).

[11]S. Greenbury and S. E. Ahnert, "The organization of biological sequences into constrained and unconstrained parts determines fundamental properties of genotype–phenotype maps", J. R. Soc. Interface **12**, 20150724 (2015).

[12]M. Weiß and S. E. Ahnert, "Using small samples to estimate neutral component size and robustness in the genotype–phenotype map of RNA secondary structure", J. R. Soc. Interface **17**, 20190784 (2020).

[13]N. S. Martin, C. Q. Camargo, and A. A. Louis, "Bias in the arrival of variation can dominate over natural selection in Richard Dawkins's biomorphs", PLOS Comput. Biol. **20**, e1011893 (2024).

[14]J. A. García-Martín, P. Catalán, S. Manrubia, and J. A. Cuesta, "Statistical theory of phenotype abundance distributions: a test through exact enumeration of genotype spaces", EPL **123**, 28001 (2018).

[15]S. Manrubia and J. A. Cuesta, "Distribution of genotype network sizes in sequence-to-structure genotype–phenotype maps", J. R. Soc. Interface **14**, 20160976 (2017).

[16]M. Weiß and S. E. Ahnert, "Phenotypes can be robust and evolvable if mutations have non-local effects on sequence constraints", J. R. Soc. Interface **15**, 20170618 (2018).

[17]V. O. Pokusaeva, D. R. Usmanova, E. V. Putintseva, L. Espinar, K. S. Sarkisyan, A. S. Mishin, et al., "An experimental assay of the interactions of amino acids from orthologous sequences shaping a complex fitness landscape", PLoS Genet. **15**, e1008079 (2019).

[18]L. Di Bari, M. Bisardi, S. Cotogno, M. Weigt, and F. Zamponi, "Emergent time scales of epistasis in protein evolution", PNAS **121**, e2406807121 (2024).

[19]A. J. Morrison, D. R. Wonderlick, and M. J. Harms, "Ensemble epistasis: thermodynamic origins of non-additivity between mutations", Genetics **219**, iyab105 (2021).
